# *Bifidobacterium infantis* associates with T cell immunity in human infants and is sufficient to enhance antigen-specific T cells in mice

**DOI:** 10.1126/sciadv.ade1370

**Published:** 2023-12-08

**Authors:** Donald D. Nyangahu, Anna-Ursula Happel, Jerome Wendoh, Agano Kiravu, Yuli Wang, Colin Feng, Courtney Plumlee, Sara Cohen, Bryan P. Brown, Danijel Djukovic, Tariq Ganief, Melanie Gasper, Daniel Raftery, Jonathan M. Blackburn, Nancy L. Allbritton, Clive M. Gray, Jisun Paik, Kevin B. Urdahl, Heather B. Jaspan

**Affiliations:** ^1^ Center for Global Infectious Disease Research, Seattle Children’s Research Institute, Seattle, WA, USA.; ^2^ Institute of Infectious Diseases and Molecular Medicine, Department of Pathology, Division of Immunology, University of Cape Town, Cape Town, South Africa.; ^3^ Department of Bioengineering, University of Washington, Seattle, WA, USA.; ^4^ Northwest Metabolomics Research Center, University of Washington, Seattle, WA, USA.; ^5^ Institute of Infectious Diseases and Molecular Medicine, Department of Integrative Biomedical Sciences, Division of Chemical and Systems Biology, University of Cape Town, Cape Town, South Africa.; ^6^ Biomedical Research Institute, Division of Molecular Biology and Human Genetics, Stellenbosch University, Cape Town, South Africa.; ^7^ Department of Comparative Medicine, University of Washington, Seattle, WA, USA.; ^8^ Department of Pediatrics, School of Medicine, University of Washington, Seattle WA, USA.

## Abstract

Bacille Calmette-Guerin (BCG) vaccine can elicit good T_H_1 responses in neonates. We hypothesized that the pioneer gut microbiota affects vaccine T cell responses. Infants who are HIV exposed but uninfected (iHEU) display an altered immunity to vaccination. BCG-specific immune responses were analyzed at 7 weeks of age in iHEU, and responses were categorized as high or low. *Bifidobacterium longum* subsp. *infantis* was enriched in the stools of high responders, while *Bacteroides thetaiotaomicron* was enriched in low responders at time of BCG vaccination. Neonatal germ-free or SPF mice orally gavaged with live *B. infantis* exhibited significantly higher BCG-specific T cells compared with pups gavaged with *B. thetaiotaomicron. B. infantis* and *B. thetaiotaomicron* differentially affected stool metabolome and colonic transcriptome. Human colonic epithelial cells stimulated with *B. infantis* induced a unique gene expression profile versus *B. thetaiotaomicron*. We thus identified a causal role of *B. infantis* in early-life antigen-specific immunity.

## Teaser

Bifidobacterium infantis in the gut correlates with BCG response in humans and enhances antigen specific T cells in mice.

## INTRODUCTION

The gut microbiota is a crucial determinant of various processes in the human body including metabolism (*1*) and immune development (*2*). In infants, the composition of the pioneer microbiota is largely determined by the mother (*3*). Consequently, factors that perturb the maternal microbiota during pregnancy and breastfeeding may potentially affect offspring microbiota (*4*). There is emerging observational evidence linking the gut microbiota to vaccine immunogenicity (*5*, *6*). A positive association between the abundance of *Bifidobacterium* early in infancy with CD4 T cell proliferation to purified protein derivative (PPD) and tetanus toxoid at 2 years has been observed (*7*). However, no causal data linking the early gut microbiota to vaccine immunogenicity in infants exists to date.

Bacille Calmette-Guerin (BCG) is administered at birth to millions of infants worldwide, including in South Africa. Known as the prototypical neonatal T cell vaccine, it can elicit robust T helper 1 (T_H_1) cytokine responses in neonates who generally have T_H_2 skewed immunity (*8*). Following the World Health Organization (WHO) 2013 recommendations for immediate lifelong antiretroviral therapy of pregnant mothers living with HIV globally, there has been a decline in vertical transmission of HIV (*9*). However, this has led to an increasing population of infants who are HIV exposed but uninfected (iHEU) (*9*). These iHEU infants have a higher risk of infectious disease morbidity and mortality compared to infants who are HIV unexposed (iHU) (*10*), making them an important target population for vaccinations. Infants exposed to HIV exhibit increased inflammation and immune activation (*11*). Moreover, iHEU have an altered gut microbiota compared to iHU infants (*12*). Studies on cell-mediated immunity, including to BCG, are confounded by the type of immune readout, assay conditions, antigen stimulations, and age at which vaccine responses are assessed. Jones and colleagues (*13*) observed no effect of HIV exposure on infant BCG-specific T cells or secreted cytokines. However, we and others have reported altered vaccine response to BCG in iHEU in response to BCG stimulation (*14*, *15*). In addition, within both iHEU and iHU, we have observed variable BCG responses (*15*), but whether the birth gut microbiota that is present at the time of BCG administration influences vaccine-induced immunity is unknown.

To address these outstanding questions, we assessed BCG responses in iHEU at week 7 of life and binarized the infants into high versus low vaccine responders. We restricted our analyses to iHEU to avoid heterogeneity introduced by HIV exposure, and because these infants have a high infectious disease morbidity, they thus require interventions to improve their immunity. To identify key gut bacteria influencing vaccine response in iHEU, we profiled the gut microbiota of high versus low BCG responders early in life when the vaccine is administered. Last, we used both a germ-free (GF) and a specific pathogen–free (SPF) neonatal mouse model to study the causative relationships between gut microbiota at birth and BCG vaccine response. We hypothesized that an aberrant gut microbiota in iHEU early in life influences immune ontogeny and vaccine immunogenicity.

## RESULTS

### T cell response to BCG are variable in infants exposed to HIV

**Fig. 1.**
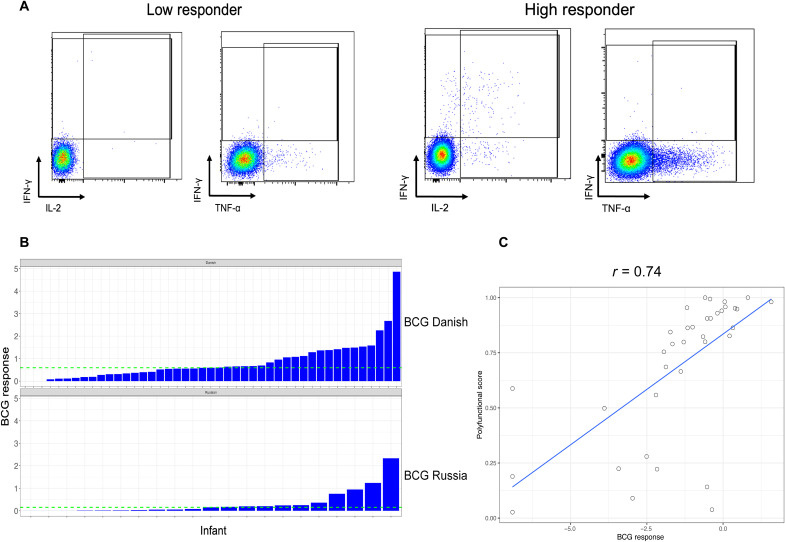
BCG response varies in HIV-exposed uninfected infants at postnatal week 7. (**A**) Representative flow plot showing CD4^+^ T cell cytokine expression following stimulation with BCG in a whole-blood assay. CD4^+^ IL-2^+^ cells depicted by blue overlay. (**B**) Spectrum of BCG vaccine responses by BCG strain in 7-week-old HIV-exposed infants measured as any Boolean combination of CD4^+^ T cells positive for IFN-γ or TNF-α or IL-2 in FlowJo. Infants were categorized into HRs or LRs around the median BCG response (green dotted line). (**C**) Correlation of polyfunctional scores generated from COMPASS analysis and BCG response. Data shown are from 66 HIV-exposed infants.

BCG recall responses were measured at 7 weeks of age after neonatal vaccination in 66 iHEUs, the age closest to the peak BCG response (*16*). iHEU displayed highly variable responses, which were measured as the frequency of CD4^+^ T cells producing any cytokine [interferon-γ (IFN-γ), interleukin-2 (IL-2), or tumor necrosis factor–α (TNF-α); Fig. 1A]. Some infants displayed high CD4^+^ cytokine production following stimulation [high polyfunctionality (Fig. 1A, right)], while others had low polyfunctionality (Fig. 1A, left). BCG responses ranged from none to 4.86% of T cells (Fig. 1B and fig. S1). This wide spectrum of responses was observed regardless of BCG strain used for vaccination (Danish or Russian). To probe these findings further, we binarized BCG responses into high responders (HRs) versus low responders (LRs) around the median response (median of 0.594% for BCG-Danish and 0.153% for BCG-Russian). Infants with BCG responses above the median (green line in Fig. 1B) were classified as HRs, while those below were categorized as LRs. COMPASS polyfunctionality analysis on a subset of infants (*17*) demonstrated a strong positive correlation (*r* = 0.74) between total T_H_1 cytokine response and polyfunctionality score (Fig. 1C). There were no differences in median gestational age, maternal age, birth weight, or most recent maternal CD4 count between HRs versus LRs. However, there was a significant association between infant sex and vaccine response (table S1).

### High versus low BCG responders have different gut microbiota composition and differentially affect immune cell populations in infant mice

**Fig. 2.**
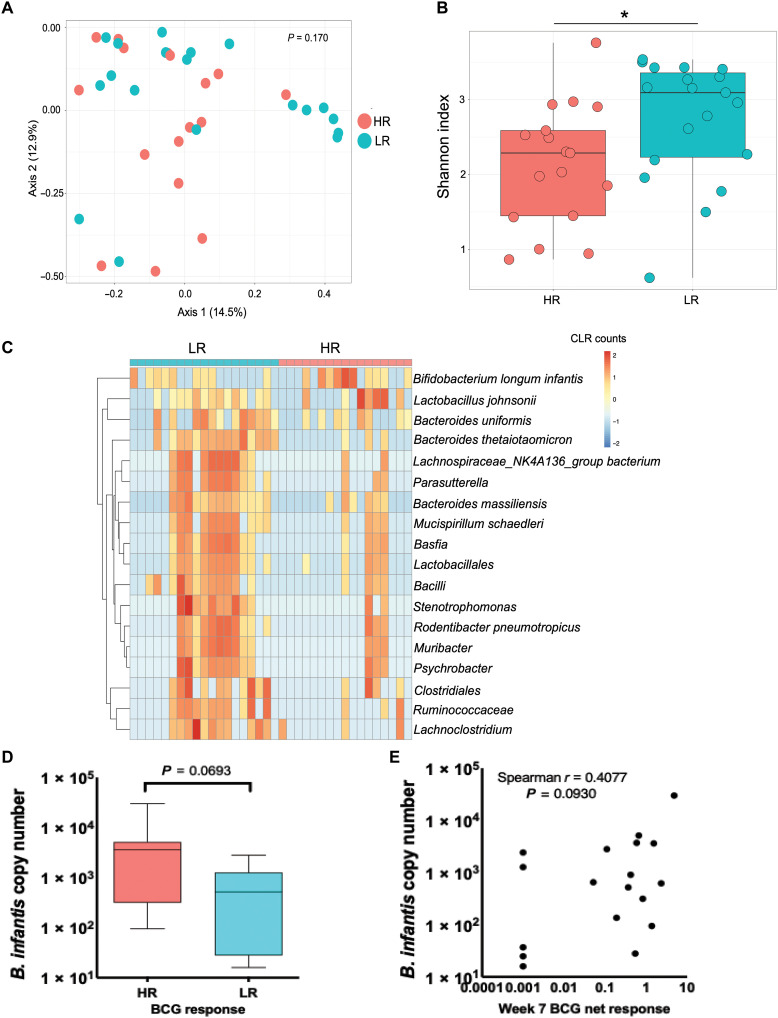
The gut microbiota differs between HRs and LRs in the first week of life. (**A**) PCA by Bray-Curtis dissimilarity. (**B**) Microbial alpha diversity by Shannon index. (**C**) Heatmap showing centered log-ratio (CLR) transformed values of differentially abundant taxa after merging taxa at the lowest annotation by metagenomeSeq. (**D**) qPCR quantification of *B. longum infantis* in infant stool in the first week of life in HR versus LR infants. (**E**) Correlation of abundance of *B. longum infantis* with BCG vaccine response.

To investigate whether variations in BCG response in infants were correlated with differences in gut microbiota at the time of vaccination, we profiled the gut microbiota from iHEU in the first week of life, closest to the time of BCG vaccination, by deep sequencing of the 16*S* ribosomal RNA (rRNA) gene V6 hypervariable region. Thirty-six infants (19 LR and 17 HR) passed our quality and sequence read depth filtering and had matching BCG response data at week 7. From these, we observed a total of 1148 amplicon sequence variants (ASVs) across the entire dataset. There was no clustering of HR versus LR stool microbial communities in a principal components analysis (PCA) by Bray-Curtis dissimilarity metric (permutational multivariate analysis of variance, *P* = 0.170; Fig. 2A). LR infants displayed significantly higher alpha diversity as measured by Shannon index compared to HR infants (*P* = 0.034; Fig. 2B). Next, we used metagenomeSeq (*18*) to identify differentially abundant taxa between HR and LR groups after merging taxa at the lowest taxonomic annotation. *Bifidobacterium longum* subspecies *infantis* was present at significantly higher differential abundance in stool of HR compared to LR infants after adjusting for birth seasonality (*P*_adj_ < 0.001; Fig. 2C and table S2). On the other hand, multiple taxa, including *Bacteroides thetaiotaomicron*, were present at significantly higher differential abundance in LR compared to HR stool (*P*_adj_ < 0.001; Fig. 2C and table S2). Quantitative polymerase chain reaction (qPCR) on DNA isolated from stools of a subset of infants with sufficient remaining DNA (*n* = 18) showed *B. infantis* to be more abundant in the HR infant stools (*P* = 0.069; Fig. 2D), and there was a weak positive correlation between copy number of *B. infantis* by qPCR and BCG responses in these infants (*P* = 0.093, Spearman *r* = 0.407; Fig. 2E), although not significant likely because of sample size.

Next, we assessed whether the infant gut microbiota influences immune development using a GF mouse model. We orally gavaged stools from HR or LR iHEU into neonatal GF mice. We selected stools from HR iHEU that had the highest BCG response and those from LR iHEU with the lowest BCG response for animal experiments. Each experiment used a pair of human infant stool (one from a HR and one from a LR infant), and two independent experiments with different infant stool pairs were conducted. Swiss-Webster (SW) neonatal GF pups were orally gavaged with either HR stool, LR stool, or phosphate-buffered saline (PBS) at day 7 of life and euthanized at day 10 (fig. S2A). Immune analyses was done by flow cytometry to measure frequencies of circulating immune subsets in the spleen. There were clear experimental effects, but trends were similar regardless of experiment. There was no difference in body weights and spleen cellularity in pups across all groups (fig. S2, B and C). Proportions of Ly6Chigh monocytes were significantly increased in HR stool–gavaged pups compared to LR-gavaged pups or PBS controls (*P* = 0.032 HR versus LR; 0.003 HR versus PBS; fig. S2E), although no difference in Ly6Clow monocytes and Ly6G^+^ neutrophils was evident (fig. S2, F and G). HR- or LR-gavaged pups exhibited similar proportions of naïve CD4 T cells (CD4^+^CD44lowCD62L^+^), although both were significantly lower compared to PBS-gavaged controls (fig. S2, H and I). HR-gavaged pups displayed significantly increased frequencies of effector memory CD4 T cells (CD4^+^CD44highCD62Llow) compared to PBS controls, but there was no difference compared to LR-gavaged mice (*P* = 0.013 PBS versus HR; *P* = 0.22 LR versus HR; fig. S2J). Central memory CD4 T cells (CD4^+^CD44highCD62Lhigh) were significantly increased in HR compared to both LR and PBS (*P* = 0.002 HR versus LR; 0.001 HR versus PBS; fig. S2K). Total memory CD4 T cells (CD4^+^CD44high) were significantly higher in both HR and LR compared to PBS (fig. S2, L and M).

Together, we show that the relative abundance of specific taxa in iHEU stool around the time of vaccination may associate with BCG T cell responses measured at later time points. In addition, we show that gut microbial communities from HR versus LR iHEU cause differential development of innate and adaptive immune cells early in life in colonized GF mice.

### Specific bacterial taxa differentially affect early immunity in pups

**Fig. 3.**
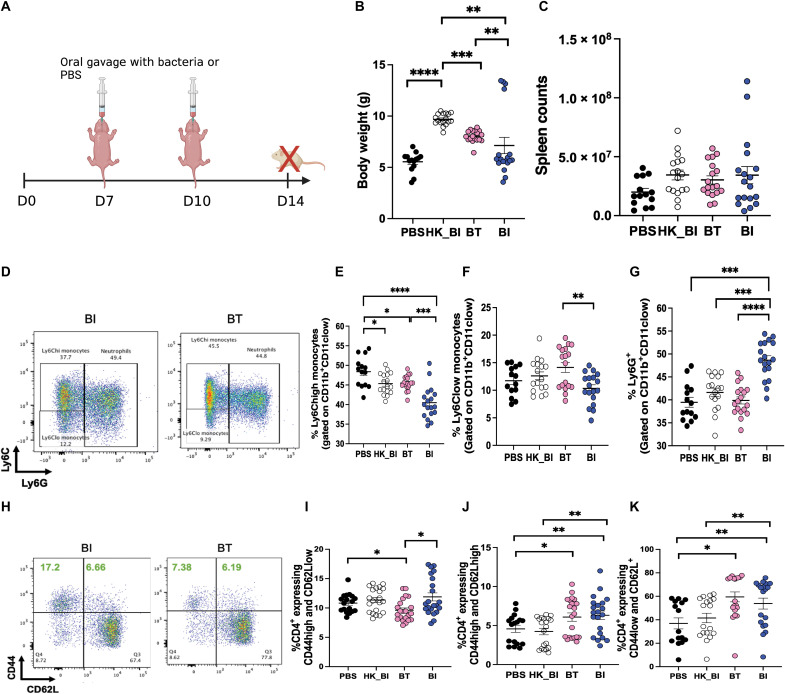
Monocolonization of neonatal GF mice with *B. longum* subspecies *infantis* or *B. thetaiotaomicron* differentially affects developing immunity in spleen. (**A**) Experimental approach: GF mice were monocolonized with either 10^8^ CFUs of *B. longum* subspecies *infantis* (BI), heat-killed BI (HK_BI), or *B. thetaiotaomicron* (BT) at day 7 of life. Control animals were colonized with PBS alone. Boost gavages of similar dosage were performed at day 10 of life. All animals were euthanized, and immunity was analyzed in spleen at day 14 of life. (**B** and **C**) Body weights and spleen counts. (**D**) Representative flow plot showing frequencies of Ly6Chigh and Ly6Clow monocytes and Ly6G^+^ neutrophils. (**E** to **G**) Frequencies of Ly6Chigh, Ly6Clow monocytes, and Ly6G^+^ neutrophils. (**H**) Representative flow plot of effector memory (CD4^+^CD44highCD62Llow), central memory (CD4^+^CD44highCD62Lhigh), and naive CD4^+^ T cells (CD4^+^CD44lowCD62L+). (**I** to **K**) Proportions of effector memory, central memory, and naive CD4^+^ T cells. Data are combined from three different experiments. Means ± SEM are plotted (*P < 0.05, **P < 0.01, ***P < 0.001, and ****P < 0.0001).

To test whether the differential bacteria identified in human infants affect immune development early in life, we used a neonatal GF or SPF murine model. We first asked whether monocolonization with *B. infantis* (higher relative abundance in HR iHEU) versus *B. thetaiotaomicron* (higher relative abundance in LR iHEU) would differentially affect developing immunity in a GF environment. Both *B. infantis* and *B. thetaiotaomicron* have previously been shown to be involved in immunoregulation and dampening inflammation in murine studies (*19*, *20*). However, the effect of these bacteria on developing immunity during infancy is unknown. To interrogate this, we monocolonized GF SW mice with 10^8^ colony-forming units (CFUs) of *B. infantis*, *B. thetaiotaomicron*, heat-killed *B. infantis*, or PBS at days 7 and 10 of life (experiment 1 in Materials and Methods; Fig. 3A). Animals that received *B. infantis* had significantly lower body weight compared to those that received *B. thetaiotaomicron* (Fig. 3B), but there was no difference in spleen cellularity (Fig. 3C). Inflammatory (Ly6Chigh) and noninflammatory monocytes (Ly6Clow) were both significantly reduced in *B. infantis* monocolonized mice compared to *B. thetaiotaomicron* monocolonized mice (Fig. 3, D to F). Frequencies of neutrophils (Ly6G^+^) were elevated in *B. infantis* versus all other groups (Fig. 3G). Effector memory CD4 T cells (CD4^+^CD44highCD62Llow) were significantly elevated in animals that received *B. infantis* compared to those that received *B. thetaiotaomicron*, but not heat-killed *B. infantis* (Fig. 3, H and I*)*. Central memory CD4 T cells (CD4^+^CD44highCD62Lhigh) were significantly elevated in all monocolonized groups compared to PBS (Fig. 3J), but naïve CD4 T cells (CD4^+^CD44lowCD62L^+^) were elevated in *B. infantis*– and *B. thetaiotaomicron*–gavaged animals versus PBS. Furthermore, live *B. infantis*–gavaged animals had significantly higher proportions of naïve CD4 T cells compared to heat-killed *B. infantis* (Fig. 3K). To evaluate whether we could detect live bacteria in monoassociated GF mice, we performed viability qPCR using *B. infantis*– or *B. thetaiotaomicron*–specific primers on day 14 stools. Propidium monoazide (PMA) qPCR has been widely used to differentiate viable from dead bacteria on the basis of the principle that PMA permeates dead cells and covalently cross-links DNA, resulting in PCR inhibition (*21*). DNA from PMA-treated stool samples obtained from live *B. infantis*–gavaged gnotobiotic mice amplified at lower *C*_t_ values compared to those from PBS- or heat-killed bacteria–gavaged mice, suggesting that the bacteria remained live in vivo (fig. S3A). A similar phenomenon was observed for *B. thetaiotaomicron* in PMA-treated samples (fig. S3B).

**Fig. 4.**
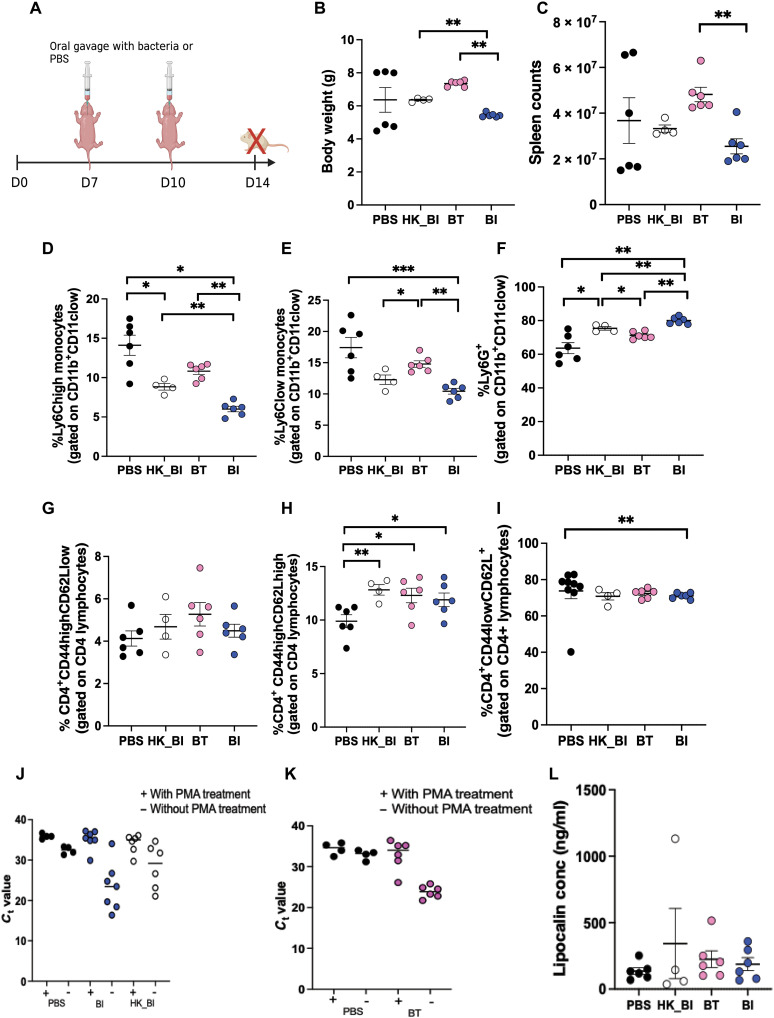
Monocolonization of neonatal SPF mice with BI or BT differentially affects developing immunity in spleen. (**A**) Experimental approach: SPF mice were orally gavaged with either 10^8^ CFUs of BI, heat-killed BI (HK_BI), or BT at day 7 of life. Control animals were colonized with PBS alone. Boost gavages of similar dosage were performed at day 10 of life. All animals were euthanized, and immunity was analyzed in spleen at day 14 of life. (**B** and **C**) Body weights and spleen counts. (**D** to **F**) Frequencies of Ly6Chigh and Ly6Clow monocytes and Ly6G^+^ neutrophils. (**G** to **I**) Proportions of effector memory, central memory, and naive CD4^+^ T cells. (**J**) *C*_t_ values with and without PMA treatment in BI-specific qPCR. (**K**) *C*_t_ values with and without PMA treatment in BT-specific qPCR. (**L**) Concentrations of lipocalin-2 in serum at day 14 of life. Data are representative of two independent experiments. Means ± SEM are plotted (*P < 0.05, **P < 0.01, and ***P < 0.001).

Next, we tested whether effects of *B. infantis* and *B. thetaiotaomicron* gavage are maintained in the presence of other gut commensals. The above described experiments were repeated under SPF conditions (experiment 2 in Materials and Methods; Fig. 4A). Like in the GF experiments, we observed significantly reduced body weight in SPF mice orally gavaged with *B. infantis* compared to *B. thetaiotaomicron* mice or heat-killed *B. infantis*–gavaged mice (Fig. 4B). Cell counts in the spleen were also significantly reduced in *B. infantis*– versus *B. thetaiotaomicron*–gavaged mice (Fig. 4C). Ly6Chigh monocytes, Ly6Clow monocytes, and neutrophils displayed similar results in SPF to what we had observed in GF (Fig. 4, D to F). Akin to GF animals, *B. infantis* group had significantly higher frequencies of central memory CD4 T cells than PBS-gavaged animals but so did heat-killed *B. infantis*–gavaged mice, which was not the case in GF experiments (Fig. 4H). Unlike in GF mice, proportions of naïve CD4 T cells were reduced in mice gavaged with *B. infantis* compared with PBS (Fig. 4I). In addition, we observed no difference in effector memory CD4 T cells between *B. infantis* and *B. thetaiotaomicron* groups (Fig. 4G). Akin to the GF experiments, we assessed bacterial viability by PMA qPCR in the SPF mice at day 14 of life (4 days after the last oral gavage). Although we could detect species-specific DNA in all live *B. infantis*, heat-killed *B. infantis*, and *B. thetaiotaomicron*, *B. infantis*, DNA amplified at similar *C*_t_ values in both live and heat-killed gavaged mouse stools in samples treated with PMA and with similar *C*_t_ values to PBS (Fig. 4, J and K). Similar results were observed for *B. thetaiotaomicron*. These results suggested that at day 14 (4 days after gavage), the time point of immune assessment, both *B. infantis* and *B. thetaiotaomicron*, which were administered live to the SPF mice, were likely not viable anymore, unlike our findings in GF mice. To determine the time course of a live gavage in the presence of a native microbiota, we performed viability qPCR at 6 and 72 hours after gavage. We detected live *B. infantis* and *B. thetaiotaomicron* at these time points (fig. S3, C to F). We noted lower *C*_t_ values in *B. thetaiotaomicron*–gavaged mice versus PBS compared to *B. infantis*, suggesting different colonization efficiencies for the two bacteria. Together, our data shows that *B. infantis* affects development of innate immune cells in a similar manner regardless of the presence of a native microbiome early in life. In addition, the effect of *B. infantis* on circulating monocytes is distinct from that of *B. thetaiotaomicron*. Moreover, we find that the impact of heat-killed *B. infantis* on CD4 T cells is influenced by other gut commensals.

To test whether the induced immune effects in spleen were due to inflammation from receipt of gavaged microbes, rather than a microbe-specific effect, we measured levels of lipocalin-2 (Lcn-2), a master mediator of inflammation shown to be elevated in serum during inflammation (*22*). We observed no difference in concentration of serum lipocalin-2 across all the groups at day 14 of life (Fig. 4L), suggesting that *B. infantis* or *B. thetaiotaomicron* bacterial gavage does not induce systemic inflammation in mice.

In summary, our results show that *B. infantis* and *B. thetaiotaomicron* differentially affect the developing immune system in neonatal mice and do not cause inflammation. Furthermore, we find that *B. infantis* induces a decrease in inflammatory monocytes compared to heat-killed *B. infantis* or *B. thetaiotaomicron* regardless of microbial environment, but the effect of live or heat-killed *B. infantis* on T cells is influenced by the native microbiota.

### *B. infantis* enhances antigen-specific T cells following BCG vaccination in GF and SPF mice

**Fig. 5.**
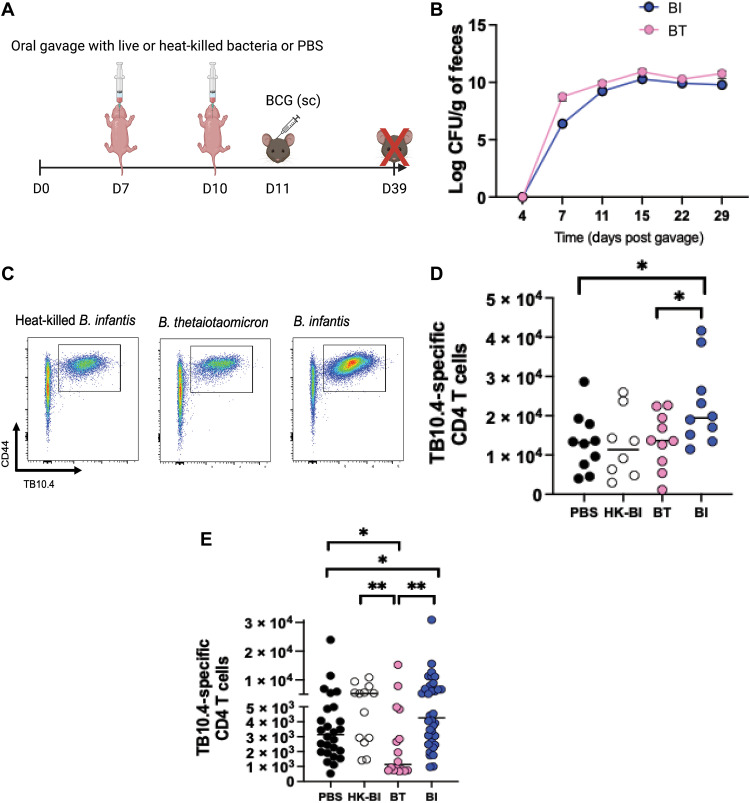
BI induces significantly higher BCG-specific CD4 T cells in recipient gnotobiotic and SPF animals. (**A**) Experimental approach: GF or SPF pups were orally gavaged with either 10^8^ CFUs of BI, BT, heat-killed BI, or PBS alone at day 7 of life. A second gavage of similar dosage was performed at day 10. All animals were immunized with 10^6^ CFUs of BCG subcutaneously at day 11 of life. Vaccine-specific responses were assessed 4-weeks after immunization. (**B**) Time course showing sequential colonization patterns of bacteria in gnotobiotic mice. CFUs were determined by plating serial dilutions of homogenized feces on agar plates. (**C**) Gating strategy to enumerate CD4^+^ TB10.4^+^ cells. (**D**) TB10.4-specific CD4 T cells in gnotobiotic mice. (**E**) TB10.4-specific CD4 T cells in SPF mice. Data are combined from at least two independent experiments. Means ± SEM are plotted (*P < 0.05 and **P < 0.01).

Correlative relationships between relative abundance of *Bifidobacterium* in stool and vaccine responses in human infants have been demonstrated (*7*). We too identified *B. infantis* relative abundance to correlate with higher BCG responses and identified *B. thetaiotaomicron* to be inversely correlated in infants (Fig. 2C). We next tested whether these bacteria were causally driving BCG response (experiment 3 in Materials and Methods; Fig. 5A). After oral gavage at days 7 and 10 into GF mice and BCG immunization at day 11 of life, animals were euthanized 4 weeks after immunization to measure BCG-specific CD4 T cells in spleens using a TB10.4 class II tetramer. We confirmed the presence of live bacteria in monocolonized mice by culturing stools in agar from day 4 through day 29 after gavage and confirmed the identity of the bacteria by Sanger sequencing of the colonies (Fig. 5B). Mice gavaged with *B. infantis* exhibited significantly higher number of TB10.4-specific CD4 T cells versus those that had been gavaged with *B. thetaiotaomicron* (*P* = 0.037; Fig. 5, C and D). Heat-killed *B. infantis* failed to elicit a similar response to live *B. infantis* in the GF environment.

To more closely mimic real-life situations, where *B. infantis* supplementation would be administered in the presence of other gut commensals, we tested effects of *B. infantis* on BCG response using SPF pups (experiment 4 in Materials and Methods). Again, animals orally gavaged with *B. infantis* in early life displayed significantly higher number of TB10.4-specific CD4 T cells compared to those gavaged with PBS or *B. thetaiotaomicron* (*P* = 0.049 *B. infantis* versus PBS; *P* = 0.001 *B. infantis* versus *B. thetaiotaomicron*; Fig. 5E). SPF animals orally gavaged with heat-killed *B. infantis* also displayed significantly higher number of antigen-specific T cells compared to *B. thetaiotaomicron* mice (*P* = 0.007) but were not different to live *B. infantis*, suggesting that the immunomodulatory effect of heat-killed *B. infantis* can be potentiated by other gut commensals (Fig. 5E). Together, we show that *B. infantis* supplementation in early life can improve T cell vaccine responses.

### *B. infantis* and *B. thetaiotaomicron* differentially affect the intestinal metabolome

**Fig. 6.**
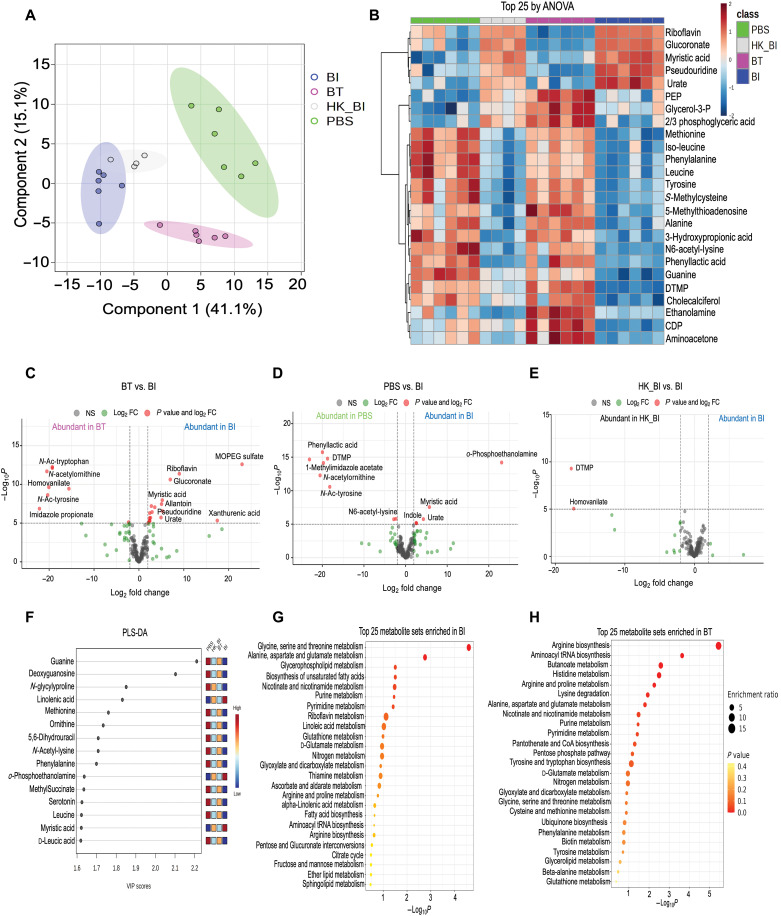
BI and BT differentially affect the stool metabolome in unvaccinated mice at day 14 of life. (**A**) PLS-DA analysis of fecal metabolite profiles of mice orally gavaged with PBS, BI, heat-killed *B. infantis* (HK_BI), or BT. (**B**) Heatmap showing supervised hierarchical clustering of the top 25 metabolites that were most different between groups by ANOVA. (**C**) Volcano plot comparing metabolite profile between BT and BI. (**D**) Volcano plot comparing metabolite profile between PBS and BI. (**E**) Volcano plot comparing metabolite profile between HK_BI and BI. (**F**) PLS-DA analysis showing the importance of fecal metabolites across all groups identified by VIP scores. (**G**) Dot plot showing enriched pathways in BI-gavaged mice. Size of the circle per pathway shows enrichment ratio, and color represents the *P* value. (**H**) Dot plot showing enriched pathways in BT-gavaged mice. Size of the circle per pathway shows enrichment ratio, and color represents the *P* value.

The gut microbiota interacts with the host by converting complex carbohydrates into readily absorbable metabolites. *B. infantis* is well known for its unique ability to metabolize human milk oligosaccharides (*23*). To investigate whether *B. infantis* or *B. thetaiotaomicron* gavage alters the host fecal metabolome, we performed targeted metabolomics of 200 aqueous metabolites in stool from SPF mice at day 14 of life (4 days after gavage; experiment 2 in Materials and Methods). We observed distinct separation of stool metabolites by gavage group by partial least squares–discriminant analysis (PLS-DA). The metabolome of *B. infantis* and heat-killed *B. infantis* groups, although distinct, clustered most closely, indicating similar metabolite profiles but both clustered distinctly from PBS and *B. thetaiotaomicron* groups (Fig. 6A). Performing pairwise differential abundance testing by limma (*24*), we identified 98 metabolites to be differentially abundant between live *B. infantis–* and *B. thetaiotaomicron–*gavaged mice, 78 metabolites between live *B. infantis* and PBS, and only 19 metabolites between *B. infantis* and heat-killed *B. infantis* groups (*P*_adj_ < 0.05; tables S3 to S5). The top 25 differentially abundant metabolites by an analysis of variance (ANOVA) displayed distinct clusters among the groups (Fig. 6B). When we filtered to include only those that had an absolute log_2_fold change ≥2, 3-methoxy-4-hydroxy-phenylglycol sulfate, riboflavin, glucuronate, myristic acid, allantoin, pseudouridine, urate, and xanthurenic acid were elevated in live *B. infantis*–gavaged mice compared to *B. thetaiotaomicron* mice (Fig. 6C). Furthermore, *o*-phosphoethanolamine, myristic acid, urate, and indole were higher in live *B. infantis*– versus PBS-gavaged mice (Fig. 6D). Supplementation with riboflavin has been shown to enhance macrophage function and dampen inflammation (*25*). Similarly, indole strengthens epithelial integrity and increases production of anti-inflammatory cytokines (*26*). There were no metabolites with log_2_fold change ≥2 in *B. infantis* versus heat-killed *B. infantis*. However, thymidine monophosphate and homovanillate were elevated in heat-killed *B. infantis* relative to live *B. infantis*–gavaged mice (Fig. 6E). PLS-DA identified linolenic acid, *o*-phosphoethanolamine, and myristic acid to exhibit high variable importance projection (VIP) scores in live *B. infantis*–gavaged mice compared to all the other groups (Fig. 6F and table S6). Linolenic acid and myristic acid are part of the milk fat globule and important for gut health (*27*). Pathway analysis revealed glycine and threonine metabolism, alanine and glutamate metabolism, glycerophospholipid metabolism, and biosynthesis of unsaturated fatty acids to be the top pathways enriched in *B. infantis*–gavaged animals (Fig. 6G and table S7). Arginine biosynthesis, aminoacyl-tRNA biosynthesis, butanoate metabolism, and histidine metabolism were the top pathways in *B. thetaiotaomicron* mice (Fig. 6H and table S8). In aggregate, these findings reveal that colonization with *B. infantis* early in life induces a fecal metabolome signature distinct from that by *B. thetaiotaomicron*. Moreover, *B. infantis* leads to enrichment of the milk-derived and tryptophan metabolite indole, which influence function of innate immune cells and epithelial barrier integrity, respectively. Furthermore, we observe very minimal differences between the metabolite profiles induced by live versus heat-killed *B. infantis* in animals with a native microbiota.

### *B. infantis* and *B. thetaiotaomicron* differentially affect the colonic transcriptome in mice

**Fig. 7.**
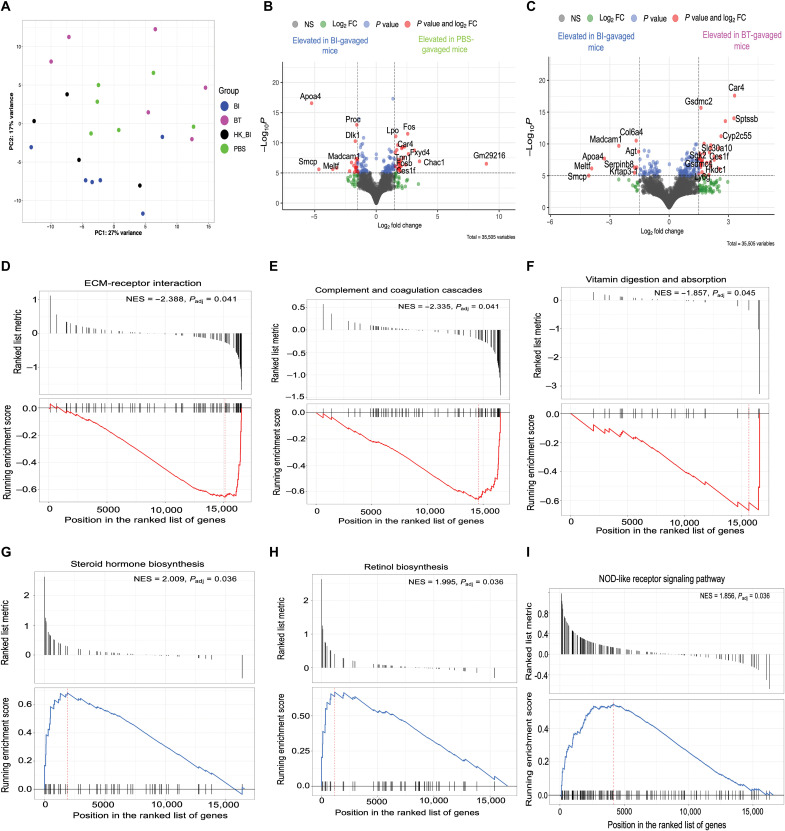
BI and BT differentially affect the colonic transcriptome in unvaccinated mice at day 14 of life. (**A**) PCA of colonic transcriptomes across groups. (**B**) Volcano plot showing DEGs between mice orally gavaged with BI versus those gavaged with PBS. (**C**) Volcano plot showing DEGs between mice orally gavaged with BI versus those gavaged with BT. (**D** to **F**) GSEA showing top pathways enriched in BI-gavaged mice compared to BT-gavaged mice. (**G** to **I**) GSEA showing top pathways enriched in BT-gavaged mice compared to BI-gavaged mice. Volcano plots were generated by EnhancedVolcano package with the fold change (FC) cutoff set at 1.5.

To interrogate the mucosal molecular signatures associated with *B. infantis* or *B. thetaiotaomicron* colonization, we assessed colonic gene expression of orally gavaged SPF mice at day 14 of life (experiment 2 in Materials and Methods). Colonic transcriptomes of live and heat-killed *B. infantis*–gavaged animals clustered distinctly from PBS or *B. thetaiotaomicron*–gavaged mice (Fig. 7A). There were 97 differentially expressed genes (DEGs) between live *B. infantis*– and *B. thetaiotaomicron*–gavaged mice. There were 83 DEGs between live *B. infantis*– and PBS-gavaged groups, and only one gene was differently expressed between live and heat-killed *B. infantis*–gavaged animals. Among others, expression of *Apoa4 Dlk1 and Madcam1* were elevated in the live *B. infantis* compared to PBS-gavaged group (Fig. 7B). Expression of *Madcam1* and *Apoa4* were also increased in live *B. infantis*– compared to *B. thetaiotaomicron*–gavaged mice, along with genes such as *Col6a4*. *Apoa4* inhibits intestinal inflammation (*28*). *Dlk1* expression is essential for normal B cell development (*29*), and *Madcam1* is an adhesion molecule involved in leukocyte homing to the gut epithelium (*30*). In contrast, the expression of *Car4,* which induces intestinal inflammation (*31*), and the neutrophil marker *Ly6g* were elevated in *B. thetaiotaomicron* compared to live *B. infantis*–gavaged mice (Fig. 7C). Only *Madcam1* was elevated in live *B. infantis* when compared to heat-killed *B. infantis*–gavaged group, suggesting live bacteria are important for gut mucosal homing. Pairwise comparisons of DEGs are shown in tables S9 and 10. Gene set enrichment analysis (GSEA) (*32*) demonstrated that extracellular matrix-receptor interaction [normalized enrichment score (NES), −2.388; *P*_adj_ = 0.041] complement and coagulation cascades (NES = −2.335, *P*_adj_ = 0.041) and vitamin digestion and absorption (NES = −1.857, *P*_adj_ = 0.045) were enriched in live *B. infantis*– compared to *B. thetaiotaomicron*–gavaged mice (Fig. 7, D to F). Other pathways like ribosome and fat digestion and absorption were also enriched in *B. infantis*–gavaged mice (table S11). Steroid hormone biosynthesis (NES = 2.009, *P*_adj_ = 0.036), retinol metabolism (NES = 1.995, *P*_adj_ = 0.036), and nonobese diabetic mice (NOD)–like receptor signaling (NES = 1.856, *P*_adj_ = 0.036) were enriched in *B. thetaiotaomicron* groups (Fig. 7, G to I). IL-17 signaling pathways were also enriched in *B. thetaiotaomicron*–gavaged animals (table S11). In summary, we demonstrate that *B. infantis* and *B. thetaiotaomicron* colonization early in life induce distinct colonic gene expression profiles and metabolic pathways. Live *B. infantis* colonization leads to the up-regulation of anti-inflammatory genes, extracellular matrix interaction, and adhesion molecules that induce mucosal homing of leukocytes, while *B. thetaiotaomicron* up-regulates proinflammatory genes and IL-17 signaling pathways.

### *B. infantis* and *B. thetaiotaomicron* differentially affect the transcriptome in human colonic cells

**Fig. 8.**
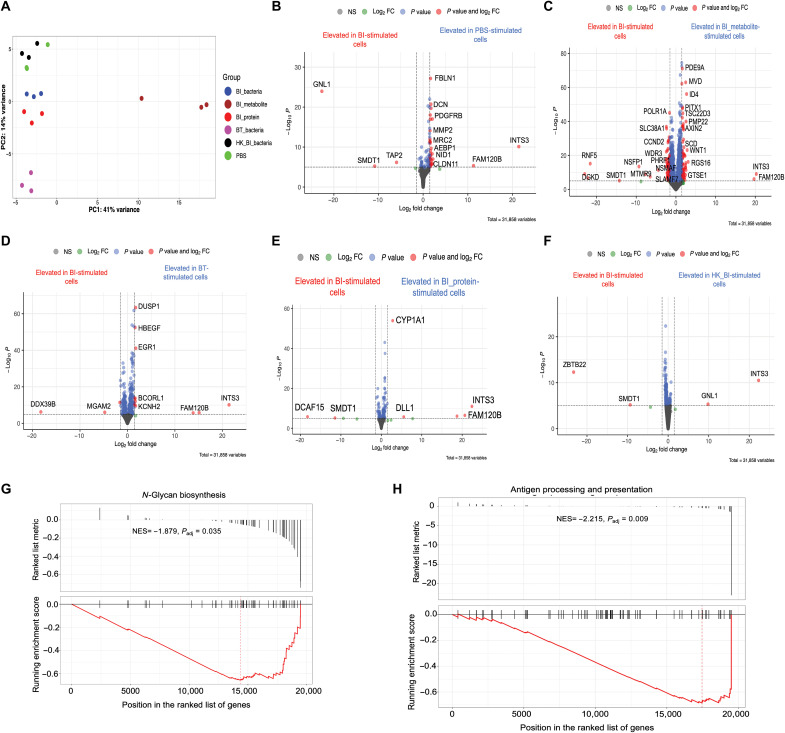
BI or its secreted products and BT differentially affect the transcriptome in stimulated human colonic epithelial cells. (**A**) PCA showing gene expression profiles in human colonic epithelial cells stimulated for 6 hours with BI, BI metabolites, BI proteins, BT, or PBS. Volcano plot showing DEGs in human colonic epithelial cells stimulated under different conditions: (**B**) BI versus PBS. (**C**) BI versus BI metabolites. (**D**) BI versus BT. (**E**) BI versus BI proteins. (**F**) BI versus heat-killed BI. (**G**) Enriched pathway in BI-stimulated cells by GSEA compared to BT-stimulated cells. (**H**) Enriched pathway in BI-stimulated cells compared to BI metabolite–stimulated cells. Volcano plots were generated by EnhancedVolcano package with the fold change cutoff set at 1.5.

Having established that *B. infantis* and *B. thetaiotaomicron* affect gene expression profiles in murine colons in vivo, we next assessed how these bacteria may interact with their canonical human host colons using an in vitro culture model. Although we found *B. infantis* to induce a distinct fecal metabolome, which may be the mechanistic link between bacteria-immune system cross-talk, secreted bacteria products other than metabolites can also drive distinct immunological effects (*33*). To enhance our mechanistic understanding of how *B. infantis* and its secreted by-products may affect human host response, we included a *B. infantis* metabolite fraction and *B. infantis* protein fraction, as well as live bacterial cultures. We expanded human colonic epithelial cells using a recently developed monolayer culture format based on a collagen-hydrogel scaffold (*34*). This system is advantageous over the organoid model, as it allows for the apical and basal side of the epithelium to be exposed, enhancing the robustness of observed findings (*35*). Once colonic epithelial cells were mature and fully differentiated, we stimulated the cells with *B. infantis* or *B. thetaiotaomicron* live bacteria, *B. infantis* metabolite fraction, *B. infantis* protein fraction, or PBS control for 6 hours and then analyzed the gene expression profile (experiment 5 in Materials and Methods). In a PCA, gene expression clustered by stimulation condition (Fig. 8A). *B. infantis* metabolites elicited a distinct gene expression signature from all other groups on PC1 (Fig. 8A). Pairwise DESeq2 comparisons showed 184 DEGs between *B. infantis* metabolite– and *B. infantis* metabolite–stimulated cells, 10 DEGs between *B. infantis* protein– and *B. infantis* protein–stimulated cells, 15 DEGs between *B. infantis* and *B. thetaiotaomicron* groups, 3 DEGs between *B. infantis* and heat-killed *B. infantis*, and 37 DEGs between *B. infantis* and PBS. This suggested that *B. infantis*–secreted proteins are likely to drive the majority of the response compared to secreted metabolites. Furthermore, we observed 252 DEGs between *B. infantis* metabolite– and PBS-stimulated cells, 51 DEGs between *B. thetaiotaomicron* and PBS, 65 DEGs between heat-killed *B. infantis* and PBS groups, and 23 DEGs between *B. infantis* proteins and PBS-stimulated cells. *TAP2* gene, which displayed elevated expression in *B. infantis* versus PBS (Fig. 8B), is involved in the antigen presentation process (*36*). *SMDT1* expression was elevated in *B. infantis*–stimulated cells compared to PBS, *B. infantis* metabolites, *B. infantis* protein, or heat-killed *B. infantis* (Fig. 8, C, E, and F). This gene regulates calcium intake in the mitochondria, and its up-regulation is associated with the inhibition of programmed death-ligand 1 (PD-L1) expression in T cells (*37*). Across all groups following pairwise comparisons with *B. infantis*–stimulated cells, we observed a down-regulation of *INTS3* and *FAM120B* genes in cells treated with *B. infantis* compared to all other conditions. These genes have been implicated in DNA damage repair and fat cell differentiation, respectively. *MGAM2*, which may be involved in mediating pattern recognition receptor signaling (*38*), and *DDX39B*, which mediates immune tolerance (*39*), were elevated in *B. infantis*– compared to *B. thetaiotaomicron*–stimulated colonic cells (Fig. 8D). Analyses of pathways by GSEA showed *N*-glycan biosynthesis to be enriched in *B. infantis*–stimulated cells compared to cells stimulated by *B. thetaiotaomicron* (NES = −1.879, *P*_adj_ = 0.035; Fig. 8G). Pathway analysis showed antigen processing, and presentation was enriched in *B. infantis* cells versus *B. infantis* metabolite cells (NES score = −2.215, *P*_adj_ = 0.009; Fig. 8H). Together, we find that *B. infantis* leads to the up-regulation of genes involved in antigen processing and presentation and fostering immune tolerance in human colonic epithelial cells cultured in vitro. Moreover, *B. infantis*–secreted proteins and not metabolites induce a molecular signature that is similar to live bacteria.

## DISCUSSION

Neonates are highly susceptible to infection and respond poorly to most vaccines. Our findings demonstrate that the gut microbiota in the first few days of life influences immune development including antigen-specific T cell responsiveness to neonatal vaccination. Infants who responded well to neonatal vaccination had higher stool relative abundance of *B. infantis* and were relatively *B. thetaiotaomicron* deficient compared to poor responders. Monocolonization of GF pups with *B. infantis* or *B. thetaiotaomicron* showed that live *B. infantis* is sufficient to elicit significantly higher antigen-specific T cells after BCG immunization. The presence of live *B. infantis* was short-lived in the SPF mouse gut, but its transient presence was sufficient to improve BCG-specific T cell responses. Furthermore, colonization with *B. infantis* early in life induced a distinct colonic transcriptome and metabolome signature. The unique effect of *B. infantis* on the transcriptome extended to human colonic epithelial cells.

Previous studies have profiled vaccine responses in HIV-exposed versus unexposed infants (*40*, *41*), but none has dissected the causal role of the gut microbiota within iHEU in driving the spectrum of immune alterations, despite reports of their altered gut microbiota (*12*, *42*). Binarizing infants into HRs versus LRs allowed us to assess microbiota variation within iHEU and tease out the effects of gut microbes on immunity independent of other potential confounders such as antiretroviral post-exposure prophylaxis, which may associate with immune phenotypes. Elegant work on the human microbiota has revealed that compositional differences in bacteria communities do not necessarily translate to functional differences. To delineate the role of specific differentially abundant bacterial taxa in LR versus HR infant stool on immune development, we assessed whether *B. infantis* and *B. thetaiotaomicron* differentially influence early immune development using both GF and SPF mice. We noted consistent results for innate cells but not memory CD4 T cells both in GF and in SPF conditions, suggesting that the influence of *B. infantis* on early development of the T cell compartment may be influenced by other gut commensals. Administration of *B. infantis* led to lower proportions of inflammatory monocytes compared to *B. thetaiotaomicron* mice. Inflammatory monocytes can exacerbate inflammatory processes in the tissue (*43*), suggesting that *B. infantis* may dampen inflammation and may be consistent with previous work (*44*). In addition, *B. infantis* also affects host metabolism. *B. infantis* gavage led to lower body weight compared to other groups. Considering that pups are only consuming breast milk at this age, our data shows that *B. infantis* is able to breakdown milk compounds, consistent with its role in digesting human milk oligosaccharides. Our results are consistent with those of others that have shown an association between *Bifidobacterium* and lean weight (*45*).

A positive correlation between gut microbiota at 6 to 15 weeks of life and T cellular proliferative responses to vaccine antigens in humans at 2 years of age has been reported (*7*). Monocolonization of GF pups with live *B. infantis* caused a significant increase in antigen-specific CD4^+^ T cells versus *B. thetaiotaomicron*, PBS, or heat-killed *B. infantis. B. infantis* but not *B. thetaiotaomicron* was able to enhance antigen-specific T cells when administered in the presence of other gut commensals, suggesting a causal association between colonization with *B. infantis* early in life and antigen-specific T cell responses. There was no difference in prevaccination concentrations of serum lipocalin-2 across all groups, suggesting that the orally administered bacteria were not inducing inflammation. Furthermore, it is plausible that the anti-inflammatory environment induced by *B. infantis* may promote T_H_1 adaptive immune responses, including to BCG vaccination. Together, our data shows an impact of *B. infantis* on cell-mediated immunity following BCG and emphasizes an indispensable role of the underlying gut microbiota early in life in shaping antigen-specific responses.

*B. infantis* and *B. thetaiotaomicron* gavage in early life led to a distinct fecal metabolome and colonic transcriptome in SPF mice. *B. infantis* led to an increase in tryptophan metabolism–derived metabolites. Up-regulation of riboflavin in the *B. infantis* group suggests elevated vitamin biosynthesis or a competitive advantage of this bacteria to use environmental vitamin B2 for its growth and survival. Transcriptomic data revealed an enrichment of vitamin biosynthesis in *B. infantis*–gavaged mice. Riboflavin is also associated with control of immune cell proliferation and differentiation (*46*), physiological processes that are likely enriched in *B. infantis*–gavaged mice. Enrichment of indole and xanthurenic acid in *B. infantis*–gavaged mice suggests elevated tryptophan metabolism and potential for enhancement of intestinal barrier function and anti-inflammatory activity in the gut (*26*), consistent with previous work (*44*). Transcriptomics revealed an enrichment of pathways associated with fat absorption and digestion in *B. infantis* mice, supporting the idea of elevated breakdown or utilization of myristic acid, which is one of the most abundant fatty acids in milk fat (*47*). Myristic acid has also been shown to have a unique immunomodulatory effect in murine models (*48*). Imidazole propionate was enriched in *B. thetaiotaomicron*–gavaged mice, indicating elevated histidine utilization by the gut microbiota and potential for low-grade inflammation and impaired glucose metabolism (*49*).

Consistent with the elevation of metabolites involved in mucosal immune programming, our transcriptomics data revealed an increased expression of *Madcam1,* which regulates leukocyte trafficking, in colons of *B. infantis* mice. Other genes including *Apoa4*, which mediate anti-inflammatory responses, were also elevated in *B. infantis*–gavaged animals. Pathways associated with immune development and interaction of immune cells were enriched in *B. infantis*, emphasizing a critical role of *B. infantis* in shaping mucosal immunity in early life and beyond. On the other hand, IL-17 signaling was repressed in *B. infantis* mice compared to *B. thetaiotaomicron*–gavaged animals. These findings are consistent with previous work, which showed *B. infantis* to inhibit T_H_17 T cell polarization (*50*). In contrast, *B. thetaiotaomicron*–gavaged mice exhibited an enrichment of proinflammatory pathways including NOD-like receptor signaling, indicative of elevated inflammation due to immune recognition of bacterial antigens. *B. infantis*–derived metabolites induced a distinct transcriptome in human colonic epithelial cells compared to live *B. infantis* cells. However, the *B. infantis* protein fraction elicited a gene expression profile similar to that of live *B. infantis*, suggesting that secreted proteins may mediate the effect of live *B. infantis* on vaccine response.

Unexpectedly, our metabolomics and transcriptomics data revealed minimal differences in the metabolome and gene expression induced by live and heat-killed *B. infantis* in SPF murine colons in vivo and human colonic epithelial cells in vitro. Heat-killed *B. infantis* given to SPF mice also led to enhanced antigen-specific T cells following vaccination, suggesting that the immune-mediated effects of *B. infantis* may be independent of bacterial viability when administered in a nonsterile environment early in life, as it would be in human infants. Gene expression profile and metabolomic profile remained distinct from that of *B. thetaiotaomicron*, suggesting that certain conserved molecular patterns in live or heat-killed *B. infantis* are recognized in a similar manner by the host triggering a beneficial immune effect during vaccination. Together, our data suggest that a surface protein of *B. infantis* may have these immunomodulatory properties. It is worth mentioning that gut colonization and engraftment efficiencies of these bacteria may be variable between mice and humans. Therefore, the effects described herein need to be tested in a clinical trial to properly assess the effect of *B. infantis* on vaccine responses. Nonetheless, our study shows a direct role for gut microbiota early in life in shaping immune development and antigen-specific T cell responses. Furthermore, we identify *B. infantis* as a potential therapeutic intervention for improved T cell immunity and vaccine responsiveness in neonates. Because *B. infantis* is already available as a live biotherapeutic, future studies should examine its impact on vaccine responses in human infants.

## MATERIALS AND METHODS

### Human cohort description

Infants were recruited as part of an ongoing observational study of mother-infant pairs at the Midwife Obstetric Unit in Site B Khayelitsha, Cape Town, South Africa, the InFANT study (*15*). This study was approved by the University of Cape Town’s Human Research Ethics Committee (reference 285/2012). Inclusion and exclusion criteria and visit schedule are described elsewhere in detail (*15*). Briefly, mothers living with or without HIV are eligible for the study, and mothers and their infants are recruited shortly after birth. All mothers participating in the study provided written informed consent for themselves and their infants. Exclusive breast feeding was recommended. All mothers with HIV received antiretroviral therapy and their infants (HIV-exposed uninfected) were confirmed to be HIV negative at birth (and were termed iHEU) by PCR and at later time points following the most recent guidelines (*51*). At 0 to 2 days of life, BCG was administered to all infants according to the WHO’s Expanded Program on Immunization. Infants received intradermal Danish BCG strain (1331; Statens Serum Institut, Denmark) from April 2013 to January 2016 and thereafter Russian strain (BCG-I Moscow, Serum Institute of India, India) due to a shortage of the Danish strain worldwide. Both strains were given at 2 × 10^5^ CFUs per dose. BCG vaccine immunogenicity was measured at 7 weeks after birth from blinded iHEU stored samples and randomly selected using the following criteria: (i) born after 36 completed weeks of gestation, (ii) a birth weight greater than 2.4 kg, and (iii) stored stool collected in the first week of life.

### Clinical measurements

#### Whole-blood BCG assay to assess BCG immunogenicity in iHEU

BCG responses were assessed in infants at 7 weeks of age as previously described (*52*). Briefly, 250 μl of anticoagulated blood was incubated with *Mycobacterium bovis* BCG vaccine (12 × 10^5^ CFU/ml; Danish strain 1331, SSI, Denmark), media alone (negative control), or PHA (50 μg/ml; positive control) within an hour of phlebotomy. This was incubated at 37°C in the clinic and transported to the laboratory in portable incubators. After 6 hours, Brefeldin A (Sigma-Aldrich) was added at a final concentration of 10 μg/ml and incubated for an additional 6 hours. Thereafter, red blood cells were lysed followed by washing and staining with LIVE/DEAD violet stain (Vivid, Thermo Fisher Scientific). Cells were then cryopreserved in 10% dimethyl sulfoxide and 90% fetal bovine serum (FBS) in liquid nitrogen (LN_2_) until analysis by flow cytometry.

#### Cell staining, data analysis, and flow cytometry in iHEU

Fixed, cryopreserved cells were thawed quickly at 37°C and washed twice with Perm/Wash buffer (BD Biosciences). Cells were then incubated in Perm/Wash for 10 min before staining with antibody cocktail mix made up in 2% FBS in PBS. Cells were stained with the following antihuman monoclonal antibodies: IL-2 PE, CD8 V500, IFN-γ Alexa Fluor 700, TNF-α PE-Cy7, and Ki67 fluorescein isothiocyanate (FITC) all from BD; CD 27 PE-cy5, HLA-DR APC-cy7, CD3 and BV650 (BioLegend); CD4 PE-cy5.5 (Invitrogen); and CD45RA PE-Texas Red (Beckman Coulter). Stained cells were incubated at 4°C for 45 min. Cells were then washed twice, resuspended in 300 μl of PBS, and acquired on the LSR II flow cytometer (BD Biosciences, San Jose, CA, USA). After acquisition, compensation and data analysis was done using FlowJo v9.9 (Tree Star, Ashland, Oregon, USA). BCG response was determined as the frequency of CD4^+^ T cells producing any cytokine (IFN-γ, TNF-α, and IL-2) minus the total T_H_1 cytokine response in the media alone condition. Net BCG responses in iHEU infants were then binarized into high versus low around the median response. COMPASS, a computational tool for analyses of polyfunctionality of antigen-specific T cells (*17*), was ran on a subset of infants.

#### 16S rRNA library preparation of iHEU stool

16*S* rRNA gene sequencing libraries were prepared as previously described from infant stools collected within 7 days of birth (*53*). Briefly, DNA was extracted from stools using the PowerSoil DNA extraction kit (Mo Bio Laboratories) and concentration determined by a Quant-IT PicoGreen dsDNA HS assay kit (Invitrogen, UK). The hypervariable V6 region of the 16*S* gene was amplified using two PCR steps: The first barcoded the samples, while the second added Illumina paired-end sequencing adapters (*54*). Resulting PCR amplicons were purified and quantified, and 50 ng was pooled into a single tube. Pooled DNA was gel-purified (QIAGEN, CA). Final library concentration was determined, and paired-end sequencing was performed at The Centre for Applied Genomics at the Hospital for Sick Children in Toronto, Canada, on the Illumina HiSeq 2000 platform [100–base pair (bp) paired-end chemistry].

#### Quantitative B. longum infantis reverse transcription PCR of iHEU stool DNA

For iHEU with sufficient remaining stool bacterial DNA, we performed qPCR to determine the copy number of *B. infantis*. The reaction mixture consisted of 5 μl of 2× PowerUP SYBR Green Master Mix, each primer pair at 0.5 μM, 2 ng of template DNA, and 1 μl of molecular-grade PCR water to make a total volume of 10 μl. The primer sequences for forward and reverse primer were 5′-ATG ATG CGC TGC CAC TGT TA-3′ and 5′-CGG TGA GCG TCA ATG TAT CT-3′, respectively. Real-time PCR was performed using the Applied Biosystems QuantStudio 7 Flex Real-Time PCR System with the following PCR conditions: Uracil-DNA glycosylases (UDG) activation (50°C for 2 min), initiation at 95°C for 2 min followed by 40 cycles at 95°C for 15 s, 56°C for 15 s, and 60°C for 1 min. Melting curve analysis was performed at 95°C for 15 s, 60°C for 1 min, and 95°C for 15 s. Standard curves were made by diluting *B. infantis* DNA from 10^1^ to 10^6^ copies/μl, and results were obtained using the Applied Biosystems software.

### Mouse experimental descriptions

#### Mice

All GF animal studies were approved by the Institutional Animal Care and Use Committee (IACUC) of University of Washington (protocol 4230-02 and 4038-02). SPF animal work was approved by the IACUC of Seattle Children’s Research Institute (SCRI) under animal protocol IACUC00328. GF SW mice were purchased from the University of Washington Gnotobiotic Animal Core or Taconic and GF BALB/c mice were purchased from Taconic (Rensselaer, NY). All GF mice were bred and maintained in open-top cages with autoclaved ALPHA-dri Plus bedding (Shepherd) and fed autoclaved rodent chow (Lab Diet, St. Louis, MO) ad libitum in flexible-film isolators. GF status of mice were checked monthly by culturing fresh fecal pellets or by performing 16*S* rRNA PCR with fecal DNA. SPF C57BL/6 and BALB/c mice (6 and 8 weeks old) were purchased from the Jackson Laboratories (Bar Harbor, ME) and maintained under SPF conditions at SCRI. Fecal transfers and immunization experiments were performed in a sterilized biosafety cabinet.

#### Monocolonization of pups (experiment 1)

To test the causal effect of specific taxa on immune development early in life, 6- to 12-week-old GF SW females were mated with males for 8 days. Dams and pups were moved to isopositive cages when the pups were 3 days old. Pups were monocolonized with 10^8^ CFUs of *B. infantis*, *B. thetaiotaomicron*, heat-killed *B. infantis* (inactivated control bacteria), or PBS (abiotic control) at day 7 and day 10 of life in 40-μl volume. Monocolonized animals were euthanized at day 14, and immunity was analyzed in the spleen by flow cytometry as described below. Stool samples collected from a subset of mice were used to assess bacterial viability by PMA qPCR (see below). Two independent experiments were conducted.

#### Impact of bacteria on early immune development in the presence of other gut commensals (experiment 2)

To investigate the impact of live or heat-inactivated *B. infantis* or *B. thetaiotaomicron* on early immune development while in the presence of other gut commensals, we conducted experiment 1 above using SPF animals. For all SPF experiments, we crossed C57BL/6 females with BALB/c males. Pups were orally gavaged with 10^8^ CFUs of *B. infantis*, *B. thetaiotaomicron*, *heat-killed B. infantis*, or PBS at days 7 and 10 of life. We analyzed splenic immunity at day 14 of life as in experiment 1. In addition, we assessed the impact of the oral bacterial interventions on the stool metabolome (see below). Furthermore, we assessed the impact of *B. infantis*, *B. thetaiotaomicron*, and *heat-killed B. infantis* supplementation on the colonic transcriptome by RNA sequencing of the colons (see below). To assess whether bacteria were colonizing the mouse gut after oral administration, we performed viability PCR (PMA qPCR) in stool collected at day 14. An additional experiment was performed to assess the time course for the presence of live gavaged bacteria with stool collected at 6 and 72 hours after gavage.

#### BCG-specific CD4 T cell response in monocolonized pups (experiment 3)

To test how gut microbiota affect BCG response, we produced mixed background pups by crossing GF SW females with GF BALB/c males because tetramers to detect immune response were only available on a BALB/c background. Pups were monocolonized with 10^8^ CFUs of *B. infantis*, *B. thetaiotaomicron*, heat-killed *B. infantis*, or PBS at day 7 and day 10 of life as in experiment 1. All animals were then immunized with 10^6^ CFUs of BCG subcutaneously at day 11 and euthanized 4 weeks after immunization. We analyzed vaccine-specific CD4 T cell responses using a tetramer containing amino acids 73 to 88 of *Mycobacterium tuberculosis* (*M. tb*) TB10.4 [I-A(d), NIH Tetramer Facility]. Stools were collected from all animals longitudinally for agar plating to confirm bacterial monocolonization. Two independent experiments were performed.

#### Modulation of BCG-specific CD4 response in presence of other commensals (experiment 4)

To test the effect of *B. infantis* or *B. thetaiotaomicron* in the presence of other commensals on BCG vaccine response, we set up breeders under SPF conditions as described in experiment 2. Pups were orally gavaged with PBS or bacteria as described in experiment 2. Pups were immunized with 10^6^ CFUs of BCG at day 11, and antigen-specific CD4 T cell responses were analyzed as in experiment 3. At least three independent experiments were performed.

#### Stimulation of human colonic epithelial cells with whole bacteria or their secreted products (experiment 5)

To assess the potential effect of *B. infantis* or *B. thetaiotaomicron* or secreted bacterial products in the human gut, we stimulated human colonic epithelial cells with bacteria or their by-products. Human colonic epithelial stem cells were expanded from the transverse colon of a cadaveric donor using a monolayer culture technique as previously described (*34*). The expanded stem cells were then plated on Falcon cell culture inserts having a porous membrane (0.4-μm pore size) to form monolayers (*35*). Briefly, the inserts were coated with 1% volume of Matrigel in PBS at 37°C overnight. Inserts were rinsed twice with PBS, and epithelial cells were plated on the top compartments of the inserts in expansion media. Cells from one well of a six-well plate were dispersed into 20 different inserts (0.4 ml in the apical side and 0.8 ml in the basal side). We exchanged the media every 48 hours. To induce cell differentiation, cells were transferred to differentiation media (media without growth factors) after 6 days, and the medium was exchanged after every 48 hours. At day 12, the monolayer was fully mature and differentiated. Epithelial cells were stimulated at the apical side for 6 hours under the following conditions: PBS, *B. infantis* (2 × 10^6^ CFU per well)*, B. thetaiotaomicron* (2 × 10^6^ CFU per well), heat-killed *B. infantis* (2 × 10^6^ CFU per well), *B. infantis* metabolite fraction, and *B. infantis* protein fraction (see below for separation of these fractions).

### Bacterial preparations for oral gavage in mice

Bacterial strains were obtained from the American Type Culture Collection (ATCC) and cultured as recommended. *B. infantis* (ATCC 15697) and *B. thetaiotaomicron* (ATCC 29148) vials were rehydrated with modified reinforced clostridial and modified chopped meat medium, respectively. Bacterial contents were then transferred and cultured in Trypticase soy agar plates anaerobically at 37°C for 48 hours. Individual colonies were picked and frozen down with 50% glycerol. New bacterial cultures were set up from freezer stocks by inoculating in broth medium anaerobically at 37°C for 48 hours. Optical densities (ODs) at 600 nm of the liquid culture were determined at 12-hour intervals alongside plating serial dilutions to determine bacteria colony counts and to establish the OD-CFU relationship. Bacterial cultures were spun down at 2000*g* for 10 min, washed twice with PBS, and resuspended in PBS for oral gavage in pups (10^8^ CFUs per mouse). To calculate the CFUs in colonized mice, stools were aseptically collected and homogenized in PBS. Homogenates were diluted (1:10,000) and dilutions plated in agar. Plates were grown anaerobically at 37°C for 48 to 72 hours after which colonies were counted.

### Fecal metabolomics

Aqueous metabolites for targeted liquid chromatography–mass spectrometry (LC-MS) analysis were extracted using a protein precipitation method. Stool samples from mice were first homogenized (Next Advance Bullet Blender Gold, Troy, NY) in 200 μl of purified deionized water at 4°C using 1-mm zirconium oxide beads (Next Advance). Because the amounts of stool samples varied, we back-calculated volumes of homogenized samples needed to have an identical amount of stool samples. We transferred the back-calculated volumes to new vials; added purified deionized water to a final volume was 200 μl for rehomogenization. Methanol containing known concentrations of 6C^13^-glucose and 2C^13^-glutamic acid was added. Afterward, samples were vortexed, stored for 30 min at −20°C, sonicated (Fisher CPX3800), centrifuged at 18,000*g*, and supernatant collected. Supernatants were dried on a SpeedVac (Eppendorf Vacufuge Plus, Enfield, CT) and reconstituted in LC-matching solvent containing 3C^13^-lactate and 2C^13^-tyrosine to monitor the assay performance. Targeted LC-MS metabolite analysis was performed on a duplex–LC-MS system composed of two Shimadzu UPLC pumps, CTC Analytics PAL HTC-xt temperature-controlled auto-sampler, and AB Sciex 6500+ Triple Quadrupole MS equipped with ESI ionization source. Measured MS peaks were integrated using AB Sciex MultiQuant 3.0.3 software. The LC-MS assay targeted 361 metabolites across all major biological pathways/cycles (plus four spiked stable isotope-labeled internal standards). From 361 targeted metabolites, 200 were measured in the study cohort (plus four spiked standards). In addition to the study samples, two sets of quality control (QC) samples were used to monitor the assay performance and data reproducibility. One QC [QC(I)] was a pooled human serum sample used to monitor system performance, and the other QC [QC(S)] consisted of a pooled study sample, which was used to monitor data reproducibility and perform data normalization and drift correction. The data were well reproducible with a median CV of 4% for the QC samples.

### PMA viability qPCR

To assess whether we can differentiate live from heat-inactivated bacteria, we performed viability qPCR, a technique that uses PMA coupled with PCR. This technique has previously been used to differentiate viable from dead bacteria (*21*). Stools collected from animals from experiments 1 and 2 at day 14 were homogenized and resuspended in PBS at 10 mg/ml. Samples were spun at 2000*g* to pellet bacteria. Samples were then treated with PMA (Biotium) in the dark and incubated for 10 min with gentle rocking at room temperature (25 μM) or left untreated. Live or heat-killed *B. infantis* or *B. thetaiotaomicron* (diluted 1:10) obtained from cultures were also treated with PMA or left untreated. All PMA-treated or untreated samples were exposed to light to cross-link the dye with DNA for 15 min. Free PMA was removed by pelleting cells by centrifugation at 5000*g* for 10 min. Pellet was resuspended in 100 μl of DNA/ribonuclease-free water and DNA extracted using the PowerSoil Pro Kit (QIAGEN, MD). The qPCR assays were performed on the StepOnePlus system (Thermo Fisher Scientific). Previously published *B. infantis* primers (5-ATGATGCGCTGCCACTGTTA-3 and 5-CGGTGA GCGTCAATGTATCT-3) and *B. thetaiotaomicron* primers (5-AACAGGTGGAAGCTGCGGA-3 and 5-AGCCTCCAACCGCATCAA-3) were used for qPCR (*55*, *56*). The reaction was performed in a final volume of 20 μl consisting of 2× EvaGreen master mix (Thermo Fisher Scientific) and 0.5 μM of each primer. For *B. thetaiotaomicron*–specific qPCR, cycling conditions used were 95 5 min, 95 1 min, 60 30s, 72 30 s, and 72 5 min. For *B. infantis*–specific qPCR, 95 2 min, 95 15 s, 56 15 s, and 60 1 min were used. DNA extracted from live or heat-killed bacterial cultures was included as positive controls. *C*_t_ values were compared across all samples with or without PMA treatment.

### Preparation of bacterial metabolites and proteins from culture for cell stimulation

*B. infantis* was grown anaerobically at 37°C in chopped meat carbohydrate media and cultures harvested at the stationary phase. Bacterial cells were separated from the supernatant by centrifugation (2000*g*, 10 min). To separate bacterial proteins from metabolites, supernatant was filtered using Amicon (10 kDa) Ultra Centrifugal filter units (Millipore). Filter units with supernatants were spun at 3000*g* for 30 min. Molecules smaller than 10 kDa went through the filter (metabolites), while larger ones were retained (proteins). To deactivate any proteins in the flow through, the metabolite fraction was heated at 50°C for 10 min. Protein concentration was determined by the Bradford Assay. Protein and metabolite fractions were used for stimulation of human colonic epithelial cells.

### RNA extraction and sequencing

RNA was extracted from frozen mouse colonic samples. Samples were obtained from mice in experiment 2 at day 14 of life. Similarly, RNA was extracted from lysed human colonic epithelial cells 6 hours after stimulation (experiment 5 above). Total RNA extraction in all samples was done using the RNeasy Mini Kit (QIAGEN) according to the manufacturer’s instructions. RNA samples were stored at −80°C until use. RNA library preparations and sequencing reactions were conducted at Azenta Life Sciences (South Plainfield, NJ). Briefly, RNA concentration was determined by Qubit Fluorometer (Thermo Fisher Scientific, Waltham, MA) and quality checked using Agilent TapeStation 4200 (Agilent Technologies, Palo Alto, CA). ERCC RNA Spike-In Mix kit (catalog no. 4456740) was added to normalize total RNA according to the manufacturer’s protocol. The RNA sequencing libraries were prepared using the NEBNext Ultra II RNA Library Prep Kit for Illumina using the manufacturer’s instructions (New England Biolabs, Ipswich, MA). Briefly, mRNAs were initially enriched with Oligod(T) beads. Enriched mRNAs were fragmented for 15 min at 94°C. First strand and second strand cDNA were subsequently synthesized. cDNA fragments were end-repaired and adenylated at 3′ ends, and universal adapters were ligated to cDNA fragments, followed by index addition and library enrichment by PCR with limited cycles. The sequencing libraries were validated on the Agilent TapeStation and quantified by using Qubit 2.0 Fluorometer and by qPCR (KAPA Biosystems, Wilmington, MA). The sequencing libraries were multiplexed and clustered onto a flow cell on the Illumina NovaSeq instrument according to the manufacturer’s instructions. The samples were sequenced using a 2 × 150 bp paired-end configuration. Every sample had at least 20 million reads.

### Flow cytometry analysis of immune cells in mice

To analyze early splenic immunity in pups, T cells were stained with anti-CD3 Alexa 700, anti-CD4 PerCPcy5.5, anti-CD8 BV510, anti-CD44 FITC, and anti-CD62L BV421. For B cells, surface staining was performed with anti-CD19 PEcy7, anti-B220 FITC, anti-CD23 BV421, anti-CD21 PE, and anti-CD80 BV786. Myeloid cells were stained with anti-CD11b FITC, anti-CD11c BV510, anti-Ly6G PEcy7, anti-Ly6C PerCPcy5.5, anti-MHCII PE, anti-NK1.1 APC, and anti-B220 BV421. Samples were incubated with antibody master mix (100 μl per sample) prepared in MACS buffer (2 mM EDTA and 0.5% BSA in PBS) that included 1% Fc block for 30 min at 4°C. Cells were washed, resuspended in acquisition buffer, and acquired on the LSR II.

To measure TB10.4-specific CD4 T cells, splenocytes from BCG-immunized mice were stained with PE-conjugated MHCII tetramer [I-A(d)] containing amino acids 73 to 88 of *M. tb* TB10.4, (NIH Tetramer Facility) for 1 hour at room temperature. Tetramer-bound cells were then labeled with anti-PE magnetic beads (Miltenyi) and enriched using magnetic columns. Tetramer-bound and unbound cells were then stained with surface markers at 4°C for 30 min. Cells were resuspended in 1% formaldehyde and acquired on the LSR II flow cytometer. After acquisition, compensation and data analysis was done using FlowJo v10.7 (Tree Star, Ashland, Oregon, USA). Antigen-specific CD4 T cells were identified as CD3^+^, CD4^+^, CD8^−^, Dump^−^ (CD11b, CD11c, F4/80, and B220), and tetramer^+^ cells. Total numbers of antigen-specific cells were recorded as the sum of antigen-specific cells in the bound and unbound fractions.

### Statistical analysis

General statistical testing among or between groups were made by Kruskal-Wallis test followed by Mann-Whitney test. Data were considered statistically significant if *P* < 0.05. GraphPad Prism software (v9) was used for all analyses.

For 16*S* microbiome analyses, raw reads were demultiplexed using in-house scripts based on sample barcodes. Primers were removed by Cutadapt using default parameters. Sequences were then imported into R and processed using the DADA2 pipeline as previously described (*57*). Briefly, the pipeline performs quality checks and filtering, learns error rates, merges paired-end reads, constructs an ASVs table, removes chimeric sequences, and assigns taxonomy to ASVs using the Silva (v138 release) reference database. Downstream analyses was performed in R using phyloseq and metagenomeSeq among other routine R packages. Community diversity was assessed by alpha diversity (within sample) and beta diversity (between sample). Before differential abundance testing by metagenomeSeq, taxa were merged at the lowest taxonomic annotation. Taxa with adjusted *P* values less than 0.05 were considered significant. For differential abundance testing, we filtered taxa to include only those with fold change ≥1.5.

For RNA sequencing analyses, quality trimming was performed by fastp (*58*) to keep only sequences with a quality score higher than 20. All sequences were retained after filtering (quality >30). Reads were then aligned by salmon (*59*) to the mouse (GRCm39) or human (GRCh38) transcriptome on Ensembl to quantify transcript-level abundances. Data analyses and differential expression analyses were performed by DESeq2 using publicly available code (https://github.com/hbctraining/Intro-to-rnaseq-hpc-salmon-flipped). Genes with an adjusted *P* value <0.05 and absolute log_2_fold change ≥1.5 were considered differentially expressed between conditions. All pairwise comparisons were made between different conditions and *B. infantis* (for both mouse colons and human colon cultures). Volcano plots were obtained using EnhancedVolcano package at a log_2_fold change cutoff of 1.5. (https://bioconductor.org/packages/devel/bioc/vignettes/EnhancedVolcano/inst/doc/EnhancedVolcano.html). GSEA was performed in R using clusterProfiler package and the Kyoto Encyclopedia of Genes and Genomes gene sets with minimum gene set size set to 20.

For metabolomics data, analysis was conducted using MetaboAnalyst (v5), a web-based tool designed for metabolomics (www.metaboanalyst.ca/). Features with constant or a single value across the entire dataset were deleted. As a default, missing values were replaced with a fifth of the minimum value for each metabolite. Data were normalized by sample sum, transformed by log transformation, and scaled by mean centering and dividing by the SD of each variable. MetaboAnalyst was used to generate PLS-DA, heatmap of topmost significant metabolites following ANOVA, and metabolite set enrichment plots. The VIP score obtained by PLS-DA combined with false discovery rate *P* value from ANOVA with post hoc test was used to evaluate differentially abundant metabolites. Differential abundance testing was additionally performed in R using limma package (*24*). Volcano plots were generated using EnhancedVolcano package with a log_2_fold change cutoff of 2.

## References

[1] L. M. Cox, S. Yamanishi, J. Sohn, A. V. Alekseyenko, J. M. Leung, I. Cho, S. G. Kim, H. Li, Z. Gao, D. Mahana, J. G. Z. Rodriguez, A. B. Rogers, N. Robine, P. Loke, M. J. Blaser, Altering the intestinal microbiota during a critical developmental window has lasting metabolic consequences. Cell 158, 705–721 (2014). 25126780 10.1016/j.cell.2014.05.052 PMC4134513

[2] J. L. Round, S. K. Mazmanian, The gut microbiome shapes intestinal immune responses during health and disease. Nat. Rev. Immunol. 9, 313–323 (2009). 19343057 10.1038/nri2515 PMC4095778

[3] A. L. Dunlop, J. G. Mulle, E. P. Ferranti, S. Edwards, A. B. Dunn, E. J. Corwin, Maternal microbiome and pregnancy outcomes that impact infant health: A review. Adv. Neonatal Care 15, 377–385 (2015). 26317856 10.1097/ANC.0000000000000218 PMC4658310

[4] E. S. Gollwitzer, B. J. Marsland, Impact of early-life exposures on immune maturation and susceptibility to disease. Trends Immunol. 36, 684–696 (2015). 26497259 10.1016/j.it.2015.09.009

[5] Q. Han, W. B. Williams, K. O. Saunders, K. E. Seaton, K. J. Wiehe, N. Vandergrift, T. A. Von Holle, A. M. Trama, R. J. Parks, K. Luo, T. C. Gurley, T. B. Kepler, D. J. Marshall, D. C. Montefiori, L. L. Sutherland, M. S. Alam, J. F. Whitesides, C. M. Bowman, S. R. Permar, B. S. Graham, J. R. Mascola, P. C. Seed, K. K. A. Van Rompay, G. D. Tomaras, M. A. Moody, B. F. Haynes, HIV DNA-adenovirus multiclade envelope vaccine induces gp41 antibody immunodominance in rhesus macaques. J. Virol. 91, 1–17 (2017). 10.1128/JVI.00923-17 PMC5640856 28794027

[6] W. B. Williams, H.-X. Liao, M. A. Moody, T. B. Kepler, S. M. Alam, F. Gao, K. Wiehe, A. M. Trama, K. Jones, R. Zhang, H. Song, D. J. Marshall, J. F. Whitesides, K. Sawatzki, A. Hua, P. Liu, M. Z. Tay, K. E. Seaton, X. Shen, A. Foulger, K. E. Lloyd, R. Parks, J. Pollara, G. Ferrari, J.-S. Yu, N. Vandergrift, D. C. Montefiori, M. E. Sobieszczyk, S. Hammer, S. Karuna, P. Gilbert, D. Grove, N. Grunenberg, M. J. M. Elrath, J. R. Mascola, R. A. Koup, L. Corey, G. J. Nabel, C. Morgan, G. Churchyard, J. Maenza, M. Keefer, B. S. Graham, L. R. Baden, G. D. Tomaras, B. F. Haynes, Diversion of HIV-1 vaccine-induced immunity by gp41-microbiota cross-reactive antibodies. Science 349, aab1253 (2015). 26229114 10.1126/science.aab1253 PMC4562404

[7] M. N. Huda, S. M. Ahmad, M. J. Alam, A. Khanam, K. M. Kalanetra, D. H. Taft, R. Raqib, M. A. Underwood, D. A. Mills, C. B. Stephensen, Bifidobacterium abundance in early infancy and vaccine response at 2 years of age. Pediatrics 143, e20181489 (2019). 30674610 10.1542/peds.2018-1489 PMC6361348

[8] A. Marchant, T. Goetghebuer, M. O. Ota, I. Wolfe, S. J. Ceesay, D. De Groote, T. Corrah, S. Bennett, J. Wheeler, K. Huygen, P. Aaby, K. P. McAdam, M. J. Newport, Newborns develop a Th1-type immune response to *Mycobacterium* bovis Bacillus Calmette-Guérin vaccination. J. Immunol. 163, 2249–2255 (1999). 10438968

[9] UNAIDS, *Fact Sheet-World AIDS Day 2017. Geneva: UN Joint Programme on HIV/AIDS (UNAIDS); Programme on HIV/AIDS* (UNAIDS, 2017); doi:978-92-9173-945-5.

[10] A. L. Slogrove, T. Goetghebuer, M. F. Cotton, J. Singer, J. A. Bettinger, Pattern of infectious morbidity in HIV-exposed uninfected infants and children. Front. Immunol. 7, 164 (2016). 27199989 10.3389/fimmu.2016.00164 PMC4858536

[11] B. Lohman-Payne, B. Gabriel, S. Park, D. Wamalwa, E. Maleche-Obimbo, C. Farquhar, R. K. Bosire, G. John-Stewart, HIV-exposed uninfected infants: Elevated cord blood Interleukin 8 (IL-8) is significantly associated with maternal HIV infection and systemic IL-8 in a Kenyan cohort. Clin. Transl. Med. 7, 26 (2018). 30198049 10.1186/s40169-018-0206-5 PMC6129453

[12] J. M. Bender, F. Li, S. Martelly, E. Byrt, V. Rouzier, M. Leo, N. Tobin, P. S. Pannaraj, H. Adisetiyo, A. Rollie, C. Santiskulvong, S. Wang, C. Autran, L. Bode, D. Fitzgerald, L. Kuhn, G. M. Aldrovandi, Maternal HIV infection influences the microbiome of HIV-uninfected infants. Sci. Transl. Med. 8, 349ra100 (2016). 10.1126/scitranslmed.aaf5103 PMC5301310 27464748

[13] C. E. Jones, A. C. Hesseling, N. G. Tena-Coki, T. J. Scriba, N. N. Chegou, M. Kidd, R. J. Wilkinson, B. Kampmann, The impact of HIV exposure and maternal *Mycobacterium* tuberculosis infection on infant immune responses to Bacille Calmette-Guérin vaccination. AIDS 29, 155–165 (2015). 25535752 10.1097/QAD.0000000000000536 PMC4284011

[14] M. A. Garcia-Knight, E. Nduati, A. S. Hassan, F. Gambo, D. Odera, T. J. Etyang, N. J. Hajj, J. A. Berkley, B. C. Urban, S. L. Rowland-Jones, Altered memory T-cell responses to Bacillus Calmette-Guerin and tetanus toxoid vaccination and altered cytokine responses to polyclonal stimulation in HIV-exposed uninfected Kenyan infants. PLOS ONE 10, e0143043 (2015). 26569505 10.1371/journal.pone.0143043 PMC4646342

[15] E. B. Kidzeru, A. C. Hesseling, J.-A. S. Passmore, L. Myer, H. Gamieldien, C. T. Tchakoute, C. M. Gray, D. L. Sodora, H. B. Jaspan, In-utero exposure to maternal HIV infection alters T-cell immune responses to vaccination in HIV-uninfected infants. AIDS 19, 1421–1430 (2014). 10.1097/QAD.0000000000000292 PMC4333196 24785950

[16] A. Kiravu, S. Osawe, A.-U. Happel, T. Nundalall, J. Wendoh, S. Beer, N. Dontsa, O. B. Alinde, S. Mohammed, P. Datong, D. W. Cameron, K. Rosenthal, A. Abimiku, H. B. Jaspan, C. M. Gray, Bacille Calmette-Guérin vaccine strain modulates the ontogeny of both mycobacterial-specific and heterologous T cell immunity to vaccination in infants. Front. Immunol. 10, 2307 (2019). 31649662 10.3389/fimmu.2019.02307 PMC6793433

[17] L. Lin, G. Finak, K. Ushey, C. Seshadri, T. R. Hawn, N. Frahm, T. J. Scriba, H. Mahomed, W. Hanekom, P.-A. Bart, G. Pantaleo, G. D. Tomaras, S. Rerks-Ngarm, J. Kaewkungwal, S. Nitayaphan, P. Pitisuttithum, N. L. Michael, J. H. Kim, M. L. Robb, R. J. O’Connell, N. Karasavvas, P. Gilbert, S. C. De Rosa, M. J. M. Elrath, R. Gottardo, COMPASS identifies T-cell subsets correlated with clinical outcomes. Nat. Biotechnol. 33, 610–616 (2015). 26006008 10.1038/nbt.3187 PMC4569006

[18] J. N. Paulson, O. C. Stine, H. C. Bravo, M. Pop, Differential abundance analysis for microbial marker-gene surveys. Nat. Methods 10, 1200–1202 (2013). 24076764 10.1038/nmeth.2658 PMC4010126

[19] S. Tanabe, Y. Kinuta, Y. Saito, Bifidobacterium infantis suppresses proinflammatory interleukin-17 production in murine splenocytes and dextran sodium sulfate-induced intestinal inflammation. Int. J. Mol. Med. 22, 181–185 (2008). 18636171

[20] M. Delday, I. Mulder, E. T. Logan, G. Grant, Bacteroides thetaiotaomicron ameliorates colon inflammation in preclinical models of Crohn’s disease. Inflamm. Bowel Dis. 25, 85–96 (2019). 30215718 10.1093/ibd/izy281 PMC6290787

[21] W. Randazzo, F. López-Gálvez, A. Allende, R. Aznar, G. Sánchez, Evaluation of viability PCR performance for assessing norovirus infectivity in fresh-cut vegetables and irrigation water. Int. J. Food Microbiol. 229, 1–6 (2016). 27085970 10.1016/j.ijfoodmicro.2016.04.010

[22] M. Vijay-Kumar, C. J. Sanders, R. T. Taylor, A. Kumar, J. D. Aitken, S. V. Sitaraman, A. S. Neish, S. Uematsu, S. Akira, I. R. Williams, A. T. Gewirtz, Deletion of TLR5 results in spontaneous colitis in mice. J. Clin. Investig. 117, 3909–3921 (2007). 18008007 10.1172/JCI33084 PMC2075480

[23] B. Zabel, C. C. Yde, P. Roos, J. Marcussen, H. M. Jensen, K. Salli, J. Hirvonen, A. C. Ouwehand, W. Morovic, Novel genes and metabolite trends in *Bifidobacterium longum* subsp. *infantis* Bi-26 metabolism of human milk oligosaccharide 2′-fucosyllactose. Sci. Rep. 9, 7983 (2019). 31138818 10.1038/s41598-019-43780-9 PMC6538704

[24] M. E. Ritchie, B. Phipson, D. Wu, Y. Hu, C. W. Law, W. Shi, G. K. Smyth, Limma powers differential expression analyses for RNA-sequencing and microarray studies. Nucleic Acids Res. 43, e47 (2015). 25605792 10.1093/nar/gkv007 PMC4402510

[25] S. Dey, B. Bishayi, Riboflavin along with antibiotics balances reactive oxygen species and inflammatory cytokines and controls *Staphylococcus* aureus infection by boosting murine macrophage function and regulates inflammation. J. Inflamm. 13, 36 (2016). 10.1186/s12950-016-0145-0 PMC5126841 27932936

[26] T. Bansal, R. C. Alaniz, T. K. Wood, A. Jayaraman, The bacterial signal indole increases epithelial-cell tight-junction resistance and attenuates indicators of inflammation. Proc. Natl. Acad. Sci. U.S.A. 107, 228–233 (2010). 19966295 10.1073/pnas.0906112107 PMC2806735

[27] D. Seki, T. Errerd, L. J. Hall, The role of human milk fats in shaping neonatal development and the early life gut microbiota. Microbiome Res. Rep. 2, 8 (2023). 38047278 10.20517/mrr.2023.09 PMC10688791

[28] T. Vowinkel, M. Mori, C. F. Krieglstein, J. Russell, F. Saijo, S. Bharwani, R. H. Turnage, W. S. Davidson, P. Tso, D. N. Granger, T. J. Kalogeris, Apolipoprotein A-IV inhibits experimental colitis. J. Clin. Investig. 114, 260–269 (2004). 15254593 10.1172/JCI21233 PMC450164

[29] R. Raghunandan, M. Ruiz-Hidalgo, Y. Jia, R. Ettinger, E. Rudikoff, P. Riggins, R. Farnsworth, A. Tesfaye, J. Laborda, S. R. Bauer, Dlk1 influences differentiation and function of B lymphocytes. Stem Cells Dev. 17, 495–507 (2008). 18513163 10.1089/scd.2007.0102 PMC3189718

[30] T. Clahsen, O. Pabst, K. Tenbrock, A. Schippers, N. Wagner, Localization of dendritic cells in the gut epithelium requires MAdCAM-1. Clin. Immunol. 156, 74–84 (2015). 25464027 10.1016/j.clim.2014.11.005

[31] E. Mizoguchi, R. J. Xavier, H. C. Reinecker, H. Uchino, A. K. Bhan, D. K. Podolsky, A. Mizoguchi, Colonic epithelial functional phenotype varies with type and phase of experimental colitis. Gastroenterology 125, 148–161 (2003). 12851880 10.1016/s0016-5085(03)00665-6

[32] E. Mizoguchi, R. J. Xavier, H.-C. Reinecker, H. Uchino, A. K. Bhan, D. K. Podolsky, A. Mizoguchi, Gene set enrichment analysis: A knowledge-based approach for interpreting genome-wide expression profiles. Proc. Natl. Acad. Sci. U.S.A. 102, 15545–15550 (2005). 16199517 10.1073/pnas.0506580102 PMC1239896

[33] M. T. Henke, D. J. Kenny, C. D. Cassilly, H. Vlamakis, R. J. Xavier, J. Clardy, *Ruminococcus gnavus*, a member of the human gut microbiome associated with Crohn’s disease, produces an inflammatory polysaccharide. Proc. Natl. Acad. Sci. U.S.A. 116, 12672–12677 (2019). 31182571 10.1073/pnas.1904099116 PMC6601261

[34] S. S. Hinman, Y. Wang, R. Kim, N. L. Allbritton, In vitro generation of self-renewing human intestinal epithelia over planar and shaped collagen hydrogels. Nat. Protoc. 16, 352–382 (2021). 33299154 10.1038/s41596-020-00419-8 PMC8420814

[35] Y. Wang, R. Kim, S.-H. J. Hwang, J. Dutton, C. E. Sims, N. L. Allbritton, Analysis of interleukin 8 secretion by a stem-cell-derived human-intestinal-epithelial-monolayer platform. Anal. Chem. 90, 11523–11530 (2018). 30199234 10.1021/acs.analchem.8b02835 PMC6309958

[36] Mantel, I., Sadiq, B. A. & Blander, J. M. Spotlight on TAP and its vital role in antigen presentation and cross-presentation. Mol. Immunol. 142, 105–119 (2022). 34973498 10.1016/j.molimm.2021.12.013 PMC9241385

[37] J. Zhu, W. Zhang, J. Chang, J. Wu, H. Wu, X. Zhang, Z. Ou, T. Tang, L. Li, M. Liu, Y. Xin, Identification and validation of a mitochondria calcium uptake-related gene signature for predicting prognosis in COAD. J. Cancer 14, 741–758 (2023). 37056383 10.7150/jca.81811 PMC10088886

[38] S. Xu, Y. Feng, S. Zhao, Proteins with evolutionarily hypervariable domains are associated with immune response and better survival of basal-like breast cancer patients. Comput. Struct. Biotechnol. J. 17, 430–440 (2019). 30996822 10.1016/j.csbj.2019.03.008 PMC6451114

[39] G. Schott, M. A. Garcia-Blanco, MHC class III RNA binding proteins and immunity. RNA Biol. 18, 640–646 (2021). 10.1080/15476286.2020.1860388 PMC8163431 33280511

[40] C. E. Jones, S. Naidoo, C. De Beer, M. Esser, B. Kampmann, A. C. Hesseling, Maternal HIV infection and antibody responses against vaccine-preventable diseases in uninfected infants. JAMA 305, 576–584 (2011). 21304083 10.1001/jama.2011.100

[41] J. A. Church, S. Rukobo, M. Govha, M. P. Carmolli, S. A. Diehl, B. Chasekwa, R. Ntozini, K. Mutasa, J. H. Humphrey, B. D. Kirkpatrick, A. J. Prendergast, Immune responses to oral poliovirus vaccine in HIV-exposed uninfected Zimbabwean infants. Hum. Vaccin. Immunother. 13, 2543–2547 (2017). 28857649 10.1080/21645515.2017.1359454 PMC5703368

[42] S. Grant-Beurmann, J. Jumare, N. Ndembi, O. Matthew, A. Shutt, A. Omoigberale, O. A. Martin, C. M. Fraser, M. Charurat, Dynamics of the infant gut microbiota in the first 18 months of life: The impact of maternal HIV infection and breastfeeding. Microbiome 10, 61 (2022). 35414043 10.1186/s40168-022-01230-1 PMC9004197

[43] C. Shi, E. G. Pamer, Monocyte recruitment during infection and inflammation. Nat. Rev. Immunol. 11, 762–774 (2011). 21984070 10.1038/nri3070 PMC3947780

[44] A. M. Ehrlich, A. R. Pacheco, B. M. Henrick, D. Taft, G. Xu, M. N. Huda, D. Mishchuk, M. L. Goodson, C. Slupsky, D. Barile, C. B. Lebrilla, C. B. Stephensen, D. A. Mills, H. E. Raybould, Indole-3-lactic acid associated with Bifidobacterium-dominated microbiota significantly decreases inflammation in intestinal epithelial cells. BMC Microbiol. 20, 357 (2020). 33225894 10.1186/s12866-020-02023-y PMC7681996

[45] Z. Xu, W. Jiang, W. Huang, Y. Lin, F. K. L. Chan, S. C. Ng, Gut microbiota in patients with obesity and metabolic disorders—A systematic review. Genes Nutr. 17, 2 (2022). 35093025 10.1186/s12263-021-00703-6 PMC8903526

[46] K. Yoshii, K. Hosomi, K. Sawane, J. Kunisawa, Metabolism of dietary and microbial vitamin b family in the regulation of host immunity. Front. Nutr. 6, 48 (2019). 31058161 10.3389/fnut.2019.00048 PMC6478888

[47] S. Verruck, C. F. Balthazar, R. S. Rocha, R. Silva, E. A. Esmerino, T. C. Pimentel, M. Q. Freitas, M. C. Silva, A. G. da Cruz, E. S. Prudencio, Dairy foods and positive impact on the consumer’s health. Adv. Food Nutr. Res. 89, 95–164 (2019). 31351531 10.1016/bs.afnr.2019.03.002

[48] N. E. Hubbard, R. J. Socolich, K. L. Erickson, Dietary myristic acid alters acylated proteins in activated murine macrophages. J. Nutr. 126, 1563–1570 (1996). 8648429 10.1093/jn/126.6.1563

[49] A. Molinaro, P. B. Lassen, M. Henricsson, H. Wu, S. Adriouch, E. Belda, R. Chakaroun, T. Nielsen, P.-O. Bergh, C. Rouault, S. André, F. Marquet, F. Andreelli, J.-E. Salem, K. Assmann, J.-P. Bastard, S. Forslund, E. Le Chatelier, G. Falony, N. Pons, E. Prifti, B. Quinquis, H. Roume, S. Vieira-Silva, T. H. Hansen, H. K. Pedersen, C. Lewinter, N. B. Sønderskov; The MetaCardis Consortium, L. Køber, H. Vestergaard, T. Hansen, J.-D. Zucker, P. Galan, M.-E. Dumas, J. Raes, J.-M. Oppert, I. Letunic, J. Nielsen, P. Bork, S. D. Ehrlich, M. Stumvoll, O. Pedersen, J. Aron-Wisnewsky, K. Clément, F. Bäckhed, Imidazole propionate is increased in diabetes and associated with dietary patterns and altered microbial ecology. Nat. Commun. 11, (2020). 10.1038/s41467-020-19589-w PMC7676231 33208748

[50] B. M. Henrick, L. Rodriguez, T. Lakshmikanth, C. Pou, E. Henckel, A. Arzoomand, A. Olin, J. Wang, J. Mikes, Z. Tan, Y. Chen, A. M. Ehrlich, A. K. Bernhardsson, C. H. Mugabo, Y. Ambrosiani, A. Gustafsson, S. Chew, H. K. Brown, J. Prambs, K. Bohlin, R. D. Mitchell, M. A. Underwood, J. T. Smilowitz, J. B. German, S. A. Frese, P. Brodin, Bifidobacteria-mediated immune system imprinting early in life. Cell 184, 3884–3898.e11 (2021). 34143954 10.1016/j.cell.2021.05.030

[51] National Department of Health, *National Consolidated Guidelines for the Prevention of Mother-To-Child Transmission of HIV (PMTCT) and the Management of HIV in Children, Adolescents and Adults* (Department of Health, Republic of South Africa, 2015), pp. 1–128.

[52] W. A. Hanekom, J. Hughes, M. Mavinkurve, M. Mendillo, M. Watkins, H. Gamieldien, S. J. Gelderbloem, M. Sidibana, N. Mansoor, V. Davids, R. A. Murray, A. Hawkridge, P. A. J. Haslett, S. Ress, G. D. Hussey, G. Kaplan, Novel application of a whole blood intracellular cytokine detection assay to quantitate specific T-cell frequency in field studies. J. Immunol. Methods 291, 185–195 (2004). 15345316 10.1016/j.jim.2004.06.010

[53] D. D. Nyangahu, K. S. Lennard, B. P. Brown, M. G. Darby, J. M. Wendoh, E. Havyarimana, P. Smith, J. Butcher, A. Stintzi, N. Mulder, W. Horsnell, H. B. Jaspan, Disruption of maternal gut microbiota during gestation alters offspring microbiota and immunity. Microbiome 6, 124 (2018). 29981583 10.1186/s40168-018-0511-7 PMC6035804

[54] J. G. Caporaso, C. L. Lauber, E. K. Costello, D. Berg-Lyons, A. Gonzalez, J. Stombaugh, D. Knights, P. Gajer, J. Ravel, N. Fierer, J. I. Gordon, R. Knight, Moving pictures of the human microbiome. Genome Biol. 12, R50 (2011). 21624126 10.1186/gb-2011-12-5-r50 PMC3271711

[55] L.-J. Teng, P.-R. Hsueh, Y.-H. Huang, J.-C. Tsai, Identification of bacteroides thetaiotaomicron on the basis of an unexpected specific amplicon of universal 16S ribosomal DNA PCR. J. Clin. Microbiol. 42, 1727–1730 (2004). 15071033 10.1128/JCM.42.4.1727-1730.2004 PMC387581

[56] H.-B. Kim, E. Kim, S.-M. Yang, S. Lee, M.-J. Kim, H.-Y. Kim, Development of real-time PCR assay to specifically detect 22 *Bifidobacterium* species and subspecies using comparative genomics. Front. Microbiol. 11, 2087 (2020). 33013760 10.3389/fmicb.2020.02087 PMC7493681

[57] D. D. Nyangahu, C. R. Plumlee, B. P. Brown, C. Feng, E. Havyarimana, S. B. Cohen, K. B. Urdahl, H. B. Jaspan, Antibiotic treatment during gestation enhances susceptibility to mycobacterium tuberculosis in offspring. Microbiol. Spectr. 10, e0249122 (2022). 36314979 10.1128/spectrum.02491-22 PMC9769670

[58] S. Chen, Y. Zhou, Y. Chen, J. Gu, Fastp: An ultra-fast all-in-one FASTQ preprocessor. Bioinformatics 34, i884–i890 (2018). 30423086 10.1093/bioinformatics/bty560 PMC6129281

[59] R. Patro, G. Duggal, M. I. Love, R. A. Irizarry, C. Kingsford, Salmon provides fast and bias-aware quantification of transcript expression. Nat. Methods 14, 417–419 (2017). 28263959 10.1038/nmeth.4197 PMC5600148

