# Supplementary material for: Bifidobacterium infantis associates with T cell immunity in human infants and is sufficient to enhance antigen-specific T cells in mice
Source: Sci Adv. 2023 Dec 8;9(49):eade1370. doi: 10.1126/sciadv.ade1370 (PMC10708209; doi:10.1126/sciadv.ade1370)
Supplement: Supplementary file 1 — Legends for figs. S1 to S3 Legends for tables S1 to S11 Figs. S1 to S3 [file sciadv.ade1370_sm.pdf]

Science Advances
AAAS

Supplementary Materials for

Bifidobacterium infantis associates with T cell immunity in human infants and is sufficient to enhance antigen-specific T cells in mice

Donald D. Nyangahu *et al.*

Corresponding author: Donald D. Nyangahu, donaldnyangahu@gmail.com; Heather B. Jaspan, hbjaspan@gmail.com

Sci. Adv. **9**, eade1370 (2023)
DOI: 10.1126/sciadv.ade1370

The PDF file includes:

Legends for figs. S1 to S3
Legends for tables S1 to S11
Figs. S1 to S3

Other Supplementary Material for this manuscript includes the following:

Tables S1 to S11

Figure S1: Flow cytometry gating strategy for whole blood assay to measure BCG response in human infants. Related to **Figure 1.**

Figure S2: High versus low responder infant gut microbiota differentially impact splenic immune development in recipient gnotobiotic neonatal mice. Germ-free pups were gavaged with fecal slurry from a high responder infant (HR), low responder infant (LR) or PBS alone. Immune analyses was performed in the spleen. **(A)** Experimental schema of fecal microbiota transplantation (FMT) on to neonatal germ-free mice. **(B&C)** Body weights and spleen cellularity. **(D)** Representative flow plot showing gating strategy to determine frequencies of neutrophils (Ly6G+), Ly6Clow and Ly6Chigh monocytes in HR versus LR gavaged animals. **(E-G)** Frequencies of Ly6Chigh, Ly6Clow monocytes and Ly6G+ neutrophils. **(I)** Representative flow plot of gating for effector memory, central memory and naive CD4+ T cells **(I-K)** Proportions of naïve, effector memory and central memory CD4+ T cells. **(L-M)** Gating of total CD4 memory cells and proportions of total CD4 memory cells (CD4+CD44high). Data are shown from two experiments with each experiment utilizing stool sets from different HR and LR infants. Mean values ±SEM are plotted (*p < 0.05, **p < 0.01, ***p < 0.001).

Figure S3: Propidium Monoazide (PMA) qPCR experiments in SPF and GF mice orally gavaged with live or heat-killed bacteria. **(A)** Ct values of DNA extracted from stool with and without PMA treatment obtained from germ-free mice gavaged with *B. infantis,* heat-killed *B. infantis* or PBS. Analysis done at day 14 of life using *B. infantis* specific qPCR. **(B)** Ct values of DNA extracted from stool with and without PMA treatment obtained from germ-free mice gavaged with *B. thetaiotaomicron* or PBS. Analysis done at day 14 of life using *B. thetaiotaomicron* specific qPCR. **(C)** Ct values of DNA extracted from stool with and without PMA treatment obtained from SPF mice gavaged with *B. infantis* or PBS. Analysis done 6 hours after oral gavage using *B. infantis* specific qPCR. **(D)** Ct values of DNA extracted from stool with and without PMA treatment obtained from SPF mice gavaged with *B. thetaiotaomicron* or PBS. Analysis done 6 hours after oral gavage using *B. thetaiotaomicron* specific qPCR. (**E)** Ct values of DNA extracted from stool with and without PMA treatment in SPF mice gavaged with *B. infantis* or PBS. Analysis done 72 hours after oral gavage using *B. infantis* specific qPCR. **(F)** Ct values of DNA extracted from stool with and without PMA treatment obtained from SPF mice gavaged with *B. thetaiotaomicron* or PBS. Analysis done 72 hours after oral gavage using *B. thetaiotaomicron* specific qPCR.

Table S1: Mother and Infant characteristics. Related to **Figure 1.**

Table S2: Differentially abundant taxa in stool of high or low vaccine responders early in life. Related to **Figure 2**

Table S3: Differentially abundant metabolites between *Bifidobacterium infantis* and *Bacteroides thetaiotaomicron* gavaged mice. Related to **Figure 6**

Table S4: Differentially abundant metabolites between *Bifidobacterium infantis* and PBS gavaged mice. Related to **Figure 6**

Table S5: Differentially abundant metabolites between *Bifidobacterium infantis* and heat-killed *Bifidobacterium infantis* gavaged mice. Related to **Figure 6**

Table S6: PLS-DA analysis of metabolites across all groups ordered by variable importance projection score. Related to **Figure 6**

Table S7: Metabolic pathways enriched in *Bifidobacterium infantis* gavaged mice. Related to **Figure 6**

Table S8: Metabolic pathways enriched in *Bacteroides thetaiotaomicron* gavaged mice. Related to **Figure 6**

Table S9: Differentially expressed genes in colon between *Bifidobacterium infantis* and *Bacteroides thetaiotaomicron* gavaged mice at day 14 of life. Related to **Figure 7**

Table S10: Differentially expressed genes in colon between *Bifidobacterium infantis* and PBS gavaged mice at day 14 of life. Related to **Figure 7**

Table S11: Gene Set Enrichment Analysis (GSEA) of enriched pathways between *Bifidobacterium infantis* and *Bacteroides thetaiotaomicron* gavaged mice. Related to **Figure 7**

Figure S1

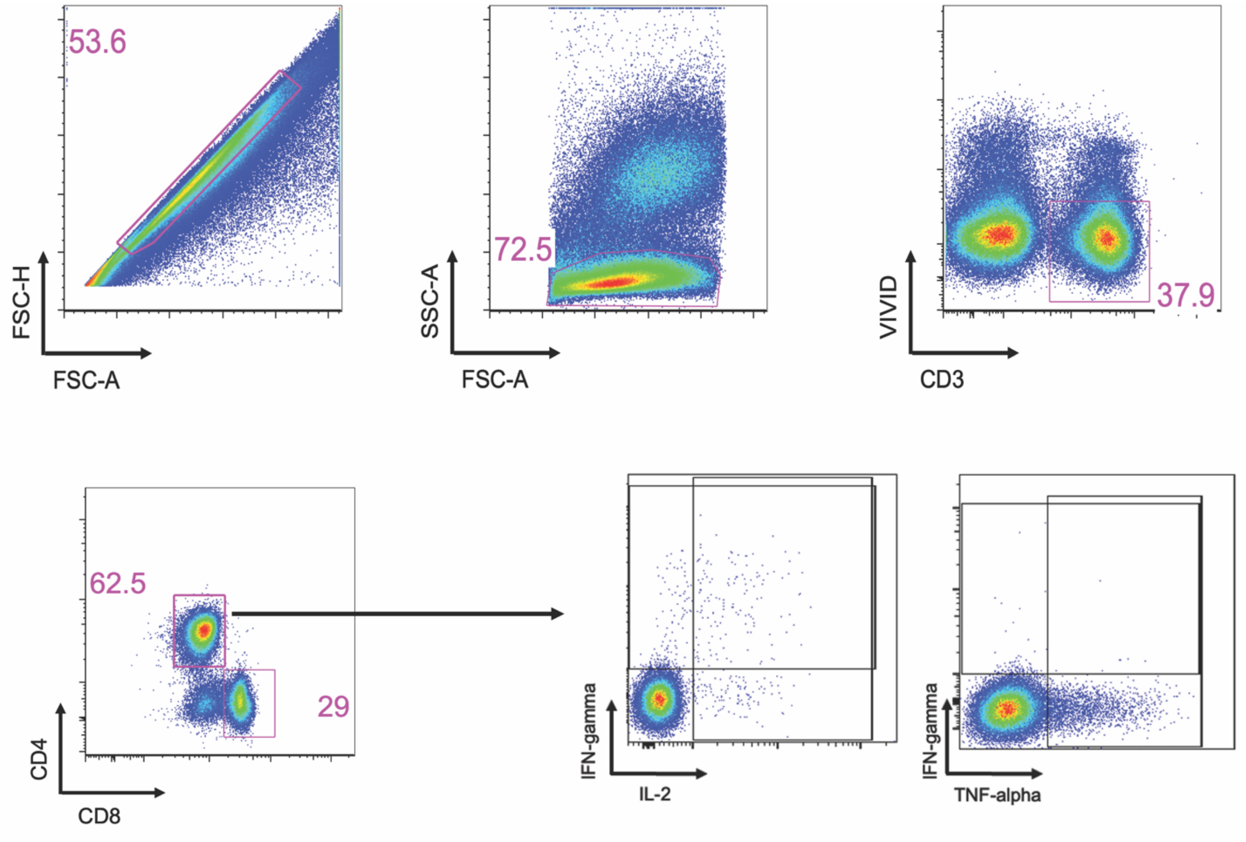

53.6
FSC-H
FSC-A
72.5
SSC-A
FSC-A
37.9
VIVID
CD3
62.5
29
CD4
CD8
IFN-gamma
IL-2
IFN-gamma
TNF-alpha

Figure S2

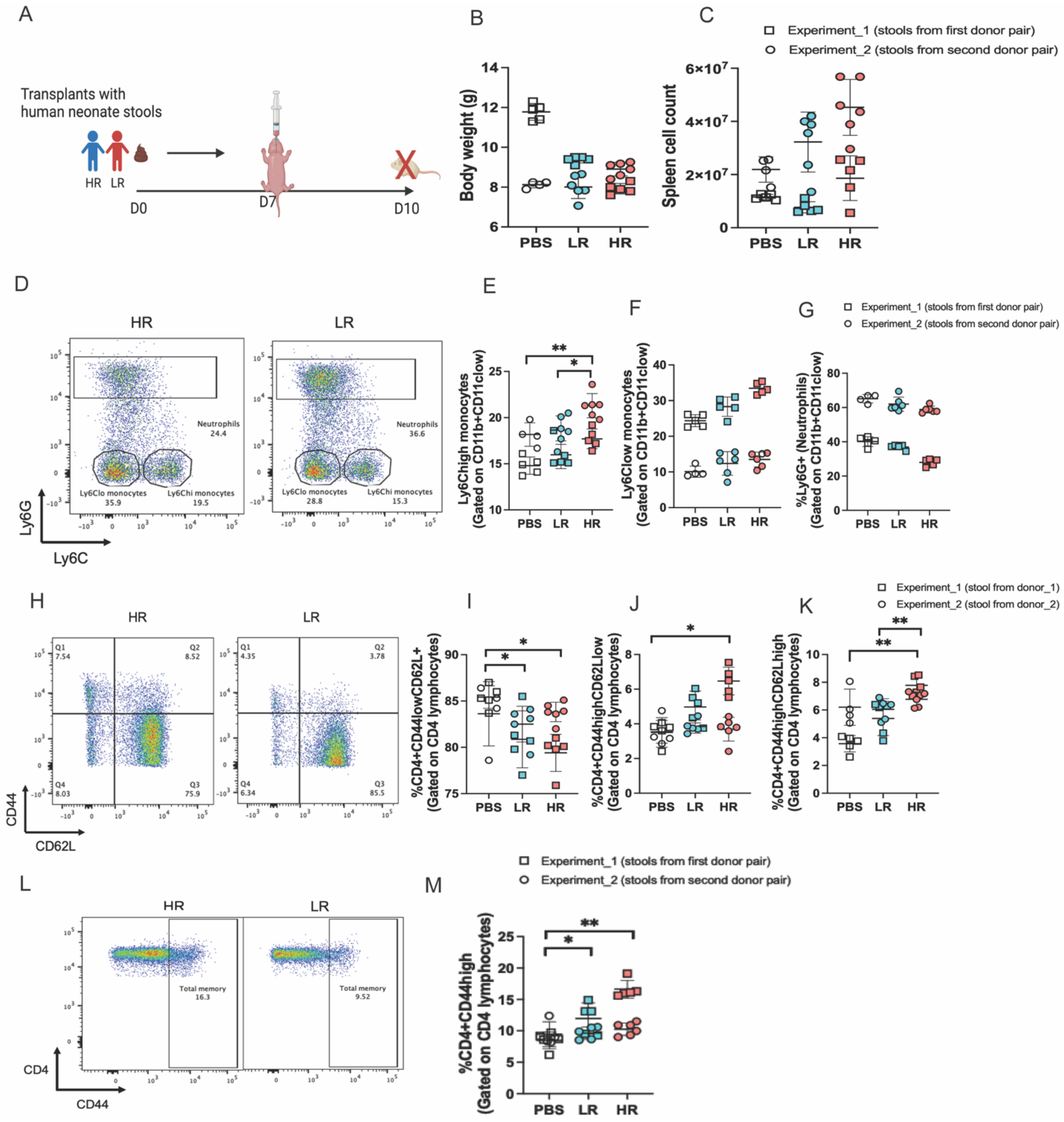

A
Transplants with human neonate stools
HR LR
D0
D7
D10
B
Body weight (g)
PBS LR HR
C
Experiment_1 (stools from first donor pair)
Experiment_2 (stools from second donor pair)
Spleen cell count
PBS LR HR
D
HR
LR
Neutrophils 24.4
Ly6Clo monocytes 35.9
Ly6Chi monocytes 19.5
Neutrophils 36.6
Ly6Clo monocytes 28.8
Ly6Chi monocytes 15.3
Ly6G
Ly6C
E
Ly6Chigh monocytes (Gated on CD11b+CD11clow)
F
Ly6Clow monocytes (Gated on CD11b+CD11clow)
G
Experiment_1 (stools from first donor pair)
Experiment_2 (stools from second donor pair)
%Ly6G+ (Neutrophils) (Gated on CD11b+CD11clow)
H
HR
LR
Q1 7.54
Q2 8.52
Q3 75.9
Q4 8.03
Q1 4.35
Q2 3.78
Q3 85.5
Q4 6.34
CD44
CD62L
I
%CD4+CD44lowCD62L+ (Gated on CD4 lymphocytes)
J
%CD4+CD44highCD62Llow (Gated on CD4 lymphocytes)
K
Experiment_1 (stool from donor_1)
Experiment_2 (stool from donor_2)
%CD4+CD44highCD62Lhigh (Gated on CD4 lymphocytes)
L
HR
LR
Total memory 16.3
Total memory 9.52
CD4
CD44
M
Experiment_1 (stools from first donor pair)
Experiment_2 (stools from second donor pair)
%CD4+CD44high (Gated on CD4 lymphocytes)
PBS LR HR

Figure S3

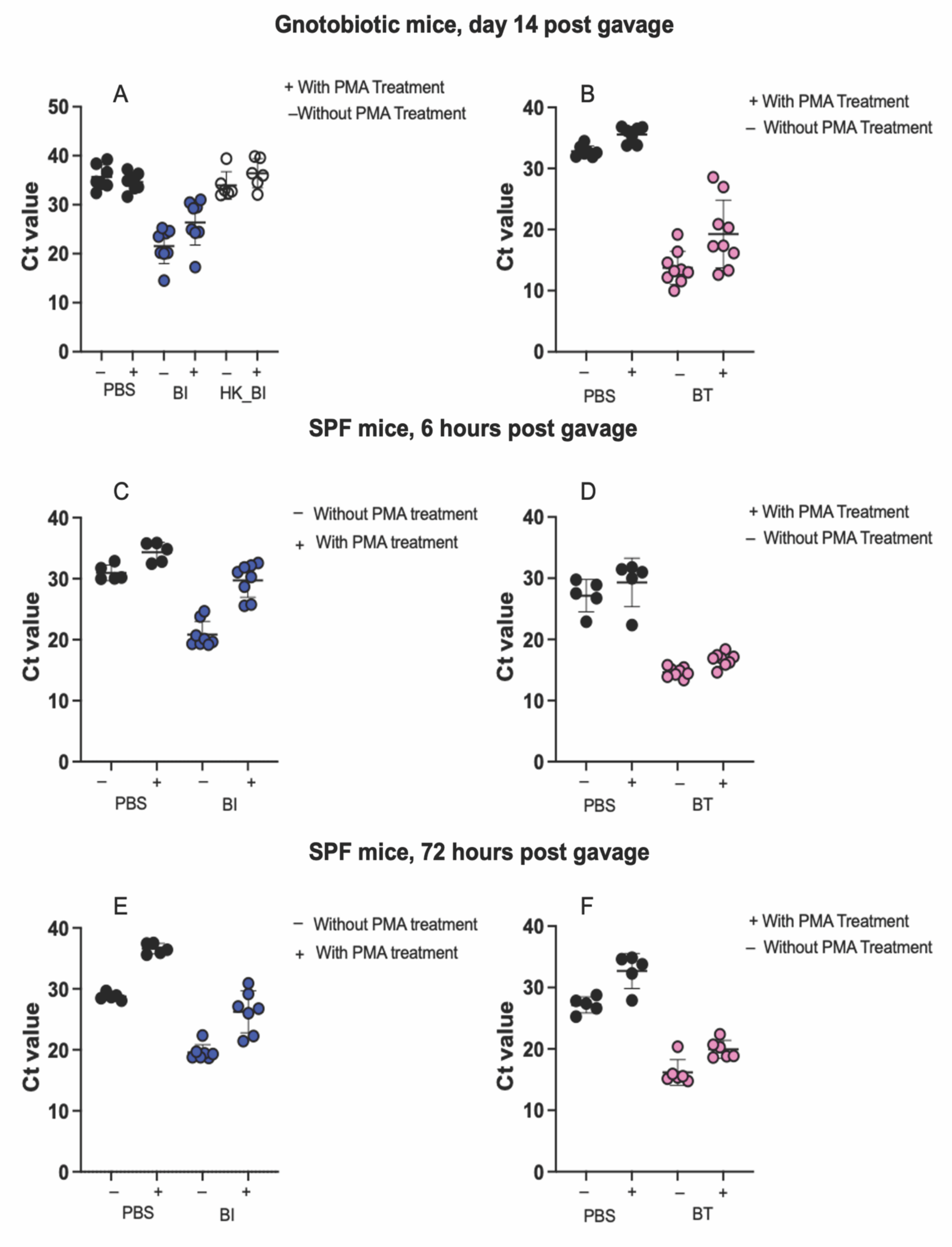
Gnotobiotic mice, day 14 post gavage
A
+ With PMA Treatment
–Without PMA Treatment
Ct value
50
40
30
20
10
0
– + – + – +
PBS
BI
HK_BI
B
+ With PMA Treatment
– Without PMA Treatment
Ct value
40
30
20
10
0
– + – +
PBS
BT
SPF mice, 6 hours post gavage
C
– Without PMA treatment
+ With PMA treatment
Ct value
40
30
20
10
0
– + – +
PBS
BI
D
+ With PMA Treatment
– Without PMA Treatment
Ct value
40
30
20
10
0
– + – +
PBS
BT
SPF mice, 72 hours post gavage
E
– Without PMA treatment
+ With PMA treatment
Ct value
40
30
20
10
0
– + – +
PBS
BI
F
+ With PMA Treatment
– Without PMA Treatment
Ct value
40
30
20
10
0
– + – +
PBS
BT
