# Supplementary material for: Bifidobacterium infantis associates with T cell immunity in human infants and is sufficient to enhance antigen-specific T cells in mice
Source: Sci Adv. 2023 Dec 8;9(49):eade1370. doi: 10.1126/sciadv.ade1370 (PMC10708209; doi:10.1126/sciadv.ade1370)
Supplement: Supplementary file 2 — Tables S1 to S11 [file sciadv.ade1370_tables_s1_to_s11.zip › ade1370_Table_S1.docx]

|  | **Low Responder Infants (LR, n=29)** | **High Responder Infants (HR, n=37)** | P-Value |
| --- | --- | --- | --- |
| Median gestational Age (Weeks); [IQR] | 39 [38—40] | 39 [38—40] | 0.295 |
| Median birth weight  (grams); [IQR] | 3200 [2660—3440] | 3130 [2820—3430] | 0.641 |
| Median maternal CD4 Count (cells/mm3); [IQR] | 470 [399.5—596.5] | 389 [272.5—539.5] | 0.209 |
| Median maternal age (years); [IQR] | 28 [24—32] | 29 [27—34] | 0.140 |
| Median number of pregnancies; [IQR] | 1 [0—2] | 1 [1—2] | 0.124 |
| Number female infants; (%) | 21 (72) | 16 (43) | 0.034 |
| Number exposed to TB; (%) | 1 (3.4) | 0 (0) | 0.439 |
| Number with maternal cough at any clinical visit; (%) | 4 (13.8) | 3 (8.1) | 0.690 |
| Number on non-antiretroviral maternal medications; (%) | 2 (6.9) | 1 (2.7) | 0.577 |
